# Dynamic risk assessment by landmark modeling of the restricted residual mean survival time

**DOI:** 10.1007/s10985-026-09731-z

**Published:** 2026-10-20

**Authors:** Yuan Zhang, Douglas E. Schaubel

**Affiliations:** 1 Division of Biostatistics, Department of Preventive Medicine, University of Tennessee Health Science Center, Memphis, TN USA; 2 Department of Biostatistics, Epidemiology and Informatics, University of Pennsylvania, Perelman School of Medicine, Philadelphia, PA USA

**Keywords:** Dependent censoring, Landmark analysis, Liver transplant, Non-proportional hazards, Restricted mean survival time, Time-varying covariate

## Abstract

Dynamic risk assessment is an important tool for healthcare providers to inform treatment selection and optimize patient outcomes. Frequent evaluation of clinical status provides invaluable information on adverse event risk and/or treatment urgency, based on the changes in predictive biomarkers. Landmark analysis is a useful approach for dynamic risk assessment that obviates the complexity of jointly modeling longitudinal covariates and time-to-event outcome. Traditional landmark methods often rely on the proportional hazards assumption and assume independent censoring. We propose novel methods to broaden the scope of landmark analysis by directly modeling the restricted residual mean survival time (RRMST) at each landmark date while accommodating dependent censoring. Advantages of RMST models include removing the assumption that the (hazard) model is correct at every time point; RMST models instead consider a single capped survival time. Moreover, investigators may prefer the area under the survival curve to the hazard function as a metric in terms of interpretability. We derive the asymptotic properties of our proposed estimators and validate their performance in finite samples through extensive simulations. We apply the proposed methods to analyze outcomes under natural end-stage liver disease (ESLD) history (i.e., in the absence of liver transplantation).

**Supplementary Information:**

The online version contains supplementary material available at https://doi.org/10.1007/s10985-026-09731-z.

## Introduction

Leveraging the increasing availability of repeatedly measured biomarkers and frequent updates of patient data (e.g., via electronic health records), dynamic risk assessment has emerged as an important tool that allows healthcare providers to tailor treatment decisions according to a patient’s medical status and urgency of treatment-initiation. Dynamic risk assessment may serve the purpose of avoiding over-treatment, maximizing quality of life, or optimizing health resource utilization. There is a great need to build refined and data-driven risk models for time-to-event endpoints in various medical fields, including oncology (Kurtz et al. 2019), end-stage renal disease (Brankovic et al. 2018), organ transplantation, infectious disease (e.g., COVID-19) (Dabbah et al. 2021), and cardiovascular disease (Damen et al. 2016).

The data structure of interest in this report features a survival time (or other time-to-event) in the presence of longitudinal biomarkers which are potential predictors of failure risk. The analytic objective is time-updated risk assessment based on frequently-updated biomarker values. Our work is motivated by the end-stage liver disease (ESLD) setting, where the preferred treatment is liver transplantation (LT). The availability of deceased donor livers for LT is vastly out-numbered by patients awaiting transplantation, necessitating an LT waiting list. One of the key goals of the liver allocation system is to minimize deaths among patients registered on the waiting list. Therefore, prioritization for LT is urgency-based, with the intention that patients predicted to die the soonest (in the absence of LT) be given the highest priority for LT. Patients move up and down the waiting list based on regularly monitored biomarkers, including creatinine, international normalized ratio (INR), serum sodium, bilirubin and dialysis status. This dynamic assessment of death risk is intended to ensure equitable allocation of available deceased donor livers. Hence, we aim to study the natural history of chronic liver disease, meaning outcomes in the absence of LT. We emphasize that the analytic objective underlying the proposed methods is risk assessment (i.e., quantification of risk factors for death in the absence of treatment) not risk prediction.

Landmark modeling (LM) (Anderson et al. 1983; van Houwelingen 2007) is gaining popularity as a practical approach to dynamic risk assessment. Essentially, a certain number of “landmark” times are defined. At each landmark time, covariates (presumably subject to frequent updates) are “frozen” to represent the patient’s current status (i.e., as of the landmark time) and are used as predictors of the residual survival time (measuring from the landmark time forward). Reasons to prefer landmark analysis over its closest competitor, joint modeling (JM) (Tsiatis and Davidian 2004; Martinussen and Scheike 2006), mostly revolve around the convenience of working with a conditional model instead of a joint model. The complexity of JM increases considerably in the presence of multiple longitudinal covariates. Moreover, mixed types of predictors, which may potentially include categorical and continuous variables, are problematic in JM. Differences between LM and JM have been extensively studied (van Houwelingen and Putter 2012; Rizopoulos et al. 2017; Bansal and Heagerty 2019). Above and beyond numerical feasibility, landmark analysis is, conceptually, better suited to the liver transplant data set which motivated our methods. In this setting, landmark analysis appropriately respects the implied hierarchy between the outcome (death) and predictors (longitudinal biomarkers). That said, landmark methods are not without limitations (Jewell and Nielsen 1993; Ferrer et al. 2018), an issue to which we return in Sect. 6.

The majority of landmark methods for survival analysis, including the partly conditional models, utilize a hazard regression model (typically, a Cox model) to quantify the association between the longitudinal predictors and the time-to-event outcome (Zheng and Heagerty 2005; Gong and Schaubel 2013). In addition, most methods assume independent censoring. Despite its long-recognized utility (Kalbfleisch and Schaubel 2023), several issues motivate the development of alternatives to hazard regression. First, the proportional hazards assumption is often violated. Second, the interpretation of covariate effects in terms of the hazard function is not always desired or understood by clinicians. Clinicians may prefer to make treatment decisions based on survival probability within a certain time window, rather than an instantaneous rate. In studying survival in the absence of treatment (also referred to as natural history), the censoring mechanism is more complicated and usually involves dependent censoring (i.e., via treatment initiation). In our motivating example, the natural history of ESLD is dependently censored by the receipt of a liver transplant.

To address the afore-listed issues specifically and to broaden the scope of landmark analysis in general, we propose landmark methods which directly model the restricted residual mean survival time (RRMST) and allow for dependent censoring. The remainder of this report is organized as follows. We describe the proposed methods, with some specific attention to model specification in Sect. 2. Asymptotic properties of the proposed estimators are derived in Sect. 3. We conduct a simulation study to evaluate the finite sample properties in Sect. 4. The proposed methods are then applied to national registry data in Sect. 5 to perform dynamic risk assessment of death in the absence of liver transplantation. We then conclude with a brief discussion in Sect. 6.

## Proposed methods

We begin by establishing the requisite notation. We then describe the proposed landmark RRMST model and detail the proposed estimation methods for modeling death in the absence of treatment (i.e., natural history). In addition, we outline various extensions to the proposed model, which may be indicated depending on the research question and data at hand. Throughout, for concreteness, we make reference to the motivating data set, for which the analysis revolves around dynamic assessment of death risk for ESLD patients wait-listed for liver transplantation. Although motivated by the ESLD setting, our methods apply more broadly to the study of time-to-event outcomes among chronic disease patients (under natural disease history).

### Notation

For the *i*th patient, *i=1,… ,n*, let $D_i$ be the treatment-free survival time (i.e., survival in the absence of treatment) which is subject to being (dependently) censored at the time of treatment initiation $T_i$ or (independently) censored at time $C_i$. The observed treatment-free follow-up time is then given by $X_i=D_i\wedge T_i\wedge C_i$, with the death indicator being $\varDelta ^D_i=I(D_i<T_i\wedge C_i)$ and the treatment indicator being $\varDelta ^T_i=I(T_i<D_i\wedge C_i)$, where $a\wedge b=\min (a,b)$ and *I(· )* represents an indicator function taking value 1 if its argument is true and 0 otherwise. At time *t*, the at-risk indicator is defined as $R_i(t)=I(X_i\geqslant t)$. A *p*-dimensional covariate vector $\boldsymbol{Z}_i(t)$ is also observed, with some elements being time-varying. For example, in the ESLD setting, important time-varying predictors include creatinine, bilirubin and albumin. We let $A_i(t)\in \{0,1\}$ denote a time-varying treatment eligibility indicator, with $A_i(t)=1$ indicating patient *i* eligible for treatment at time *t* and 0 indicating ineligible. In the liver transplant data set, treatment eligibility refers to the patient being *active* on the LT waiting list. A wait-listed patient may be *deactivated* for various reasons, including infection, illness, or resumption of alcohol consumption. These conditions are usually temporary, such that patients are expected to become active again once the conditions resolves. While inactive, a patient is not allowed to receive a deceased-donor transplant. Thus, we have a longitudinally observed covariate history $\widetilde{\boldsymbol{Z}}_i(t)=\{\boldsymbol{Z}_i(s);s\in [0,t]\}$ and treatment-eligibility history $\widetilde{A}_i(t)=\{A_i(s);s\in [0,t]\}$. The observed data for patient *i* are therefore represented by $\mathcal {O}_i=\big \{X_i,\varDelta ^D_i,\varDelta ^T_i,\widetilde{\boldsymbol{Z}}_i(X_i),\widetilde{A}_i(X_i)\big \}$. The maximum observed follow-up time is denoted as $\tau =\max \{X_1,\dots ,X_n\}$.

### Landmarking

To set up the landmarking, we first choose *K* cross-section times (calendar dates), denoted as $\text {CS}_k$, *k=1,… ,K*. In many settings amenable to landmark analysis, landmark times can be defined as (regular) follow-up times (e.g., at 0, 6, 12 months). However, in this report we distinguish between the concepts of calendar time scale and follow-up time scale because, in the ESLD setting, a deceased-donor liver offer can occur on any date, and offer dates are not aligned with any patient’s follow-up time. Moreover, patients enter the study (at wait-listing for a liver transplant) on different calendar dates, with biomarkers updated at irregular visits thereafter. The order in which the wait-listed and eligible patients are prioritized to receive a donor offer is typically determined based on the cross-sectional information available on the offer date. A patient’s decision to accept or decline a given liver offer will be influenced, in part, by the patient’s predicted prognosis in the absence of transplantation. Hence, our calendar time cross-section dates, $\text {CS}_k$, correspond to dates on which a donor offer (and hence patient decision-making) could occur.

We now define notation to represent residual time since cross-section date. Let $S_{ik}$ be the follow-up time of the *i*th patient at the *k*th cross-section, $\text {CS}_k$. The at-risk indicator at $\text {CS}_k$ for patient *i* is denoted as $R_{ik}=R_i(S_{ik})$; i.e., patient *i* has been registered on the waiting list, alive, untreated, and uncensored. The treatment-eligibility indicator at $\text {CS}_k$ for patient *i* is denoted as $A_{ik}=A_i(S_{ik})$. At each $\text {CS}_k$, we reset the time-to-event clock then measure the residual event time since the cross-section date. Hence, the landmark model is then conditional survival until at least the cross-section date. Let $D_{ik}=D_i-S_{ik}$ denote the remaining time-to-death at the *k*th cross-section; $T_{ik}=T_i-S_{ik}$ denote the remaining time-to-treatment; and $C_{ik}=C_i-S_{ik}$ denote the remaining time-to-censoring. The observed treatment-free event time and the corresponding event indicators for patient *i* at $\text {CS}_k$ are $(X_{ik},\varDelta ^D_{ik},\varDelta ^T_{ik})$, where $X_{ik}=X_i-S_{ik}$, $\varDelta ^D_{ik}=I(D_{ik}<T_{ik}\wedge C_{ik})$, and $\varDelta ^T_{ik}=I(T_{ik}<D_{ik}\wedge C_{ik})$. It follows that $R_{ik}=I(X_{ik}\geqslant 0)$, $\varDelta ^D_{ik}=\varDelta ^D_i$ and $\varDelta ^T_{ik}=\varDelta ^T_i$.

**Fig. 1:**
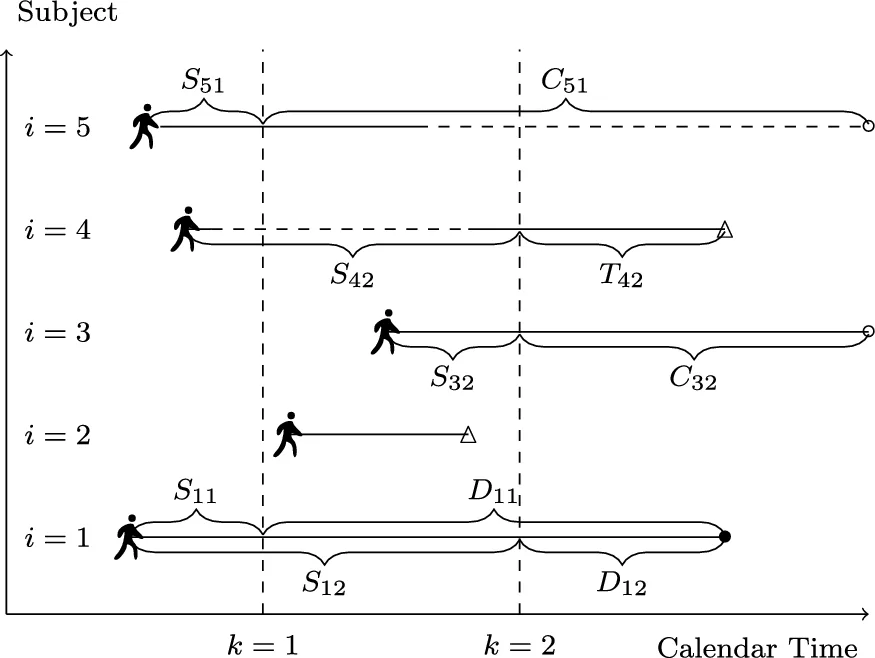
Example of landmark analysis with two cross-sections for five subjects. The human figure at the beginning of each patient journey indicates study entry, i.e., the calendar date at which the patient is approved and wait-listed for a liver donation. At the end of follow-up for each patient journey, a solid circle indicates death; a hollow circle indicates loss to follow-up, i.e., independent censoring; a hollow triangle indicates receipt of a liver transplant, i.e., dependent/informative treatment censoring. The patient progress is indicated by a line, where a solid line represents the subject at-risk of death and eligible for receiving a liver transplant, and a dashed line represents the subject at-risk of death but ineligible for the treatment

We illustrate the landmark process in Fig. 1. The example considers two cross-sections *k=1,2*. We are interested in predicting the pre-treatment risk score for subjects who are active on the wait list. Note that, for a particular cross-section date, we focus on active (transplant-eligible) patients since only such patients will be making decisions about donor liver offers. Therefore, patients who not yet enter the study; who are dead, treated or censored; or who are treatment-ineligible are excluded from the cross-sectional data obtained at each landmark. Since the landmark model is conditional on patients alive at each cross-section, the residual lifetime is of main interest. In Fig. 1, Subject 1 has nonnegative remaining death times $D_{11}$ and $D_{12}$ at $\text {CS}_1$ and $\text {CS}_2$, respectively, and therefore is included in the landmark analysis at both cross-sections. Subject 2 is not included in either cross-section because s/he enters the study after the 1 st cross-section and is treated before the 2nd cross-section. Subject 3 has remaining censoring time $C_{32}$ at $\text {CS}_2$. Subject 4 has remaining treatment time $T_{42}$ at $\text {CS}_2$. Note that, although Subject 4 is treatment-ineligible at $\text {CS}_1$, s/he is treatment-eligible at $\text {CS}_2$ and therefore should be included in the 2nd cross-sectional data. Subject 5 has remaining censoring time $C_{51}$ at $\text {CS}_1$. The stacked data therefore have five records, two at the 1 st cross-section (Subjects 1 and 5) and three at the 2nd cross-section (Subjects 1, 3, and 4).

In order to formulate the observed data in the direct RMST framework, we set *L* as a pre-specified restriction time, typically selected to be clinically meaningful (e.g., 3 months, 1 year, or 5 years). Let $Y_{ik}=X_{ik}\wedge L$ be the residual survival time truncated at *L* at the *k*th cross-section, provided that s/he is alive at the *k*th cross-section ($R_{ik}=1$). The corresponding event indicator is defined as $\varDelta ^Y_{ik}=I(D_{ik}\wedge L<T_{ik}\wedge C_{ik})$.

### Proposed landmark model

Of chief interest is the residual time survived after the *k*th cross-section. We denote the mean of this quantity, capped at *L*, by $\mu _{ik}(L)$, with

$$\begin{aligned} \mu _{ik}(L)=\mathbb {E}\big \{D_{ik}\wedge L\big |\widetilde{\boldsymbol{Z}}_i(S_{ik}),\widetilde{A}_i(S_{ik}),A_{ik}=1,D_i>S_{ik}\big \}. \end{aligned}$$

We refer to this quantity as the *retricted residual mean survival time* (RRMST). Note that other researchers have used the term conditional RMST (Yang et al. 2021). To maintain parsimony of landmark models, we assume

$$\begin{aligned} \mu _{ik}(L)=\mathbb {E}\big (D_{ik}\wedge L\big |\boldsymbol{Z}_{ik},A_{ik}=1,D_i>S_{ik}\big ), \end{aligned}$$

where $\boldsymbol{Z}_{ik}=\boldsymbol{Z}_i(S_{ik})$ represents a vector comprised of pre-specified functions of prior follow-up time, $S_{ik}$, and the covariate history observed up to $S_{ik}$. A common specification of $\boldsymbol{Z}_{ik}$ could simply be a vector of baseline covariates and time-varying covariates evaluated at the *k*th cross-section. In practice, patients may have irregular visits for covariate measurements with different intervals. If a landmark date is between two visits, then we apply the method of last observation carried forward (LOCF). Note that one could also interpolate between consecutive measurements (Schaubel et al. 2009). For ease of notation, we drop *L* in $\mu _{ik}(L)$ for the subsequent paragraphs because only one restriction time is considered for data analysis.

We specify a model for the association between the RRMST $\mu _{ik}$ and the covariates $\boldsymbol{Z}_{ik}$ *stratified* by cross-section. We develop estimation procedures for an *additive* model (Zhang and Schaubel 2024),

1 $$\begin{aligned} \mu _{ik}^A(\boldsymbol{Z}_{ik};\boldsymbol{\beta }):=\mu _{0k}+\boldsymbol{\beta }'\boldsymbol{Z}_{ik}, \end{aligned}$$

and for a *multiplicative* model (Wang et al. 2019),

2 $$\begin{aligned} \mu _{ik}^M(\boldsymbol{Z}_{ik};\boldsymbol{\beta }):=\mu _{0k}\exp (\boldsymbol{\beta }'\boldsymbol{Z}_{ik}), \end{aligned}$$

where $\boldsymbol{\beta }$ is the *p× 1* parameter vector of interest.

In our application, the landmark times are calendar dates which are set equal across patients (hence, the term “cross-section”). If patients enter the study on different calendar dates, then landmark dates do not align with any patient’s follow-up time and, hence, are not important in terms of prognosis and could be treated as nuisance parameters. Moreover, the potentially complex parametric form of landmark effects may be difficult to specify. The above two considerations motivate a stratified model. Gong and Schaubel (2017) provide a detailed explanation of the rationale of using a stratified partly conditional model. The two models Eqs. (1) and (2) are considered due to a common feature that the landmark effect, which is a nuisance parameter, can be factored out of the estimation procedure, analogous to stratified Cox regression. We discuss additional modeling considerations and extensions (which add much flexibility to Eqs.(1) and (2)) in Sect. 2.5. Note that stratification by landmark time serves to non-parametrically adjust for background calendar time effects.

### Parameter estimation

In order to construct unbiased estimating equations for $\boldsymbol{\beta }$ in the presence of a mixture of independent and dependent censoring, we apply inverse probability weighting for both independent and treatment censoring (IPCW). We assume that independent censoring time $C_i$ is conditionally independent of $D_i$ and $T_i$ given baseline covariates, and treatment time $T_i$ is dependent of $D_i$ only through the time-varying covariate process $\widetilde{\boldsymbol{Z}}_i(t)$. Suppose $C_i$ follows the Cox proportional hazards model,

3 $$\begin{aligned} \lambda _i^C(t;\boldsymbol{\theta }_C)=\lambda _0^C(t)\exp \left( \boldsymbol{\theta }_C'\boldsymbol{Z}^C_i\right) , \end{aligned}$$

where $\boldsymbol{Z}^C_i$ denotes the predictors of independent censoring, and the corresponding cumulative hazard for subject *i* at time *t* is $\varLambda ^C_i(t;\boldsymbol{\theta }_C)=\int ^t_0\lambda ^C_i(u;\boldsymbol{\theta }_C)du$. We define the inverse probability of independent censoring weights to be $W_{ik}^C=P\big (C_{ik}>Y_{ik}\big |C_i>S_{ik},X_{ik},\boldsymbol{Z}^C_i\big )^{-1}=\exp \left\{ \varLambda ^C_i(Y_{ik}+S_{ik})-\varLambda ^C_i(S_{ik})\right\}$. Essentially, the inverse weighting serves to assign relatively higher (lower) weight to subjects who are more (less) likely to be censored.

Note that, to fit model Eq. (3), we assume that $C_i$ is hazard-independent of both $T_i$ and $D_i$. Specifically, if we write $\lambda _i^C(t)= \lambda ^C(t|C_i\geqslant t,\boldsymbol{Z}^C_i)$, we assume $\lambda _i^C(t)=\lambda ^C(t|C_i\geqslant t,\boldsymbol{Z}^C_i,D_i>t,T_i>t)$.

Similarly, the probability of treatment for patients who are treatment-eligible at the *k*th cross-section, is estimated through the following Cox model,

4 $$\begin{aligned} \lambda _i^T(t;\boldsymbol{\theta }_T)=A_i(t)\lambda _0^T(t)\exp \left\{ \boldsymbol{\theta }_T'\boldsymbol{Z}^T_i(t)\right\} , \end{aligned}$$

where $\boldsymbol{Z}^T_i(t)$ denotes the potentially time-varying predictors of receiving a liver transplant, and the corresponding cumulative hazard for subject *i* at time *t* is $\varLambda ^T_i(t;\boldsymbol{\theta }_T)=\int ^t_0\lambda ^T_i(u;\boldsymbol{\theta }_T)du$. We expect the distribution of time-to-treatment to depend on time-varying covariates in our application. For example, bilirubin serves as a biomarker indicating the severity of liver disease; an elevation in serum bilirubin level signifies that the patient is in a more critical condition, with an increased likelihood of receiving a liver transplant. Note that creatinine and bilirubin are formally built into the liver allocation rules in the United States. We define the inverse probability of treatment weights to be $W_{ik}^T=P\big \{T_{ik}>Y_{ik}\big |T_i>S_{ik},X_{ik},\boldsymbol{Z}^T_i(S_{ik})\big \}^{-1}=\exp \left\{ \varLambda ^T_i(Y_{ik}+S_{ik})-\varLambda ^T_i(S_{ik})\right\}$. Other forms of treatment weights have been proposed in the literature.

With respect to the treatment censoring, to fit model Eq. (4), we assume that $T_i$ is hazard-independent of both $C_i$ and $D_i$. Specifically, if we write $\lambda _i^T(t)= \lambda ^T\left\{ t|T_i\geqslant t,\boldsymbol{Z}^T_i(t),A_i(t)\right\}$, we assume $\lambda _i^T(t)=\lambda ^T\left\{ t|T_i\geqslant t,\boldsymbol{Z}^T_i(t),A_i(t),D_i>t,C_i>t\right\}$.

Under the afore-described set-up, unbiased estimating equations for $\boldsymbol{\beta }$ and $\mu _{0k}$, *k = 1,… ,K*, are given by

5 $$\begin{aligned} \sum _{k=1}^K&\sum _{i=1}^nR_{ik}A_{ik}W_{ik}\varDelta ^Y_{ik}\boldsymbol{Z}_{ik}\left\{ Y_{ik}-\mu _{ik}(\boldsymbol{Z}_{ik};\boldsymbol{\beta })\right\} =\boldsymbol{0}, \end{aligned}$$

6 $$\begin{aligned}&\sum _{i=1}^nR_{ik}A_{ik}W_{ik}\varDelta ^Y_{ik}\left\{ Y_{ik}-\mu _{ik}(\boldsymbol{Z}_{ik};\boldsymbol{\beta })\right\} =0,\hspace{0.1in} k=1,\dots ,K, \end{aligned}$$

respectively, where $W_{ik}=W^C_{ik}W^T_{ik}$, and $\mu _{ik}(\boldsymbol{Z}_{ik};\boldsymbol{\beta })$ can take the form of $\mu _{ik}^A(\boldsymbol{Z}_{ik};\boldsymbol{\beta })$ in the additive model Eq. (1) or $\mu _{ik}^M(\boldsymbol{Z}_{ik};\boldsymbol{\beta })$ in the multiplicative model Eq. (2). $R_{ik}A_{ik}$ is the indicator that patient *i* is selected into the cross-sectional data at $\text {CS}_k$. Proof of the unbiasedness of Eqs.(5) and (6) can be found in Supplementary Information Appendix A.

Here we outline the estimation procedures using landmark RRMST modeling. First, we derive the stacked dataset for a set of landmark dates $\text {CS}_k$, *k = 1,… ,K*. That is, for each *k*,

- Calculate the follow-up time (time previously survived) for each patient at the *k*th cross-section, $S_{ik}$.
- Filter patients who are at risk, i.e., $X_i> S_{ik}$, and treatment eligible, i.e., $A_i(S_{ik})=1$;
- Compute the residual follow-up times: $X_{ik}=X_i-S_{ik}$, $Y_{ik}=X_{ik}\wedge L$, and $\varDelta ^Y_{ik}=\varDelta ^D_{ik}$ if $X_{ik}<L$ and 1 otherwise;
- Extract the covariate values $\boldsymbol{Z}_{ik}$ (using LOCF or interpolation) in the case of missing measurements at the exact time $S_{ik}$.

Second, we combine these *K* cross-sectional data sets and solve Eqs. (5) and (6) using the stacked data to obtain $\widehat{\boldsymbol{\beta }}$. Solving the *p+K* estimating equations simultaneously can be computationally challenging. Since the landmark intercepts $\mu _{0k}$, *k=1,… ,K*, are not of primary interest, we first express $\mu _{0k}$ as a function of $\boldsymbol{\beta }$ using Eq. (6) and then substitute the expression into Eq. (5) to obtain an estimator for $\boldsymbol{\beta }$. For the additive model $\mu _{ik}^A(\boldsymbol{Z}_{ik};\boldsymbol{\beta })$, a closed-form expression for $\widehat{\boldsymbol{\beta }}$ can be derived as

$$\begin{aligned} \widehat{\boldsymbol{\beta }}&=\bigg [\sum ^{K}_{k=1}\sum ^{n}_{i=1}\left\{ \boldsymbol{Z}_{ik}-\bar{\boldsymbol{Z}}_k^A(\widehat{\boldsymbol{W}}_k)\right\} ^{\otimes 2}R_{ik}A_{ik}\widehat{W}_{ik}\varDelta _{ik}^Y\bigg ]^{-1}\\&\hspace{8em}\bigg [\sum ^{K}_{k=1}\sum ^{n}_{i=1}\left\{ \boldsymbol{Z}_{ik}-\bar{\boldsymbol{Z}}_k^A(\widehat{\boldsymbol{W}}_k)\right\} R_{ik}A_{ik}\widehat{W}_{ik}\varDelta _{ik}^YY_{ik}\bigg ], \end{aligned}$$

where we define

$$\begin{aligned} \bar{\boldsymbol{Z}}_k^A(\boldsymbol{W}_k)= & \frac{\sum \limits ^{n}_{i=1}R_{ik}A_{ik}W_{ik}\varDelta ^Y_{ik}\boldsymbol{Z}_{ik}}{\sum \limits ^{n}_{i=1}R_{ik}A_{ik}W_{ik}\varDelta ^Y_{ik}}, \end{aligned}$$

with $\boldsymbol{W}_k=(W_{1k},\dots ,W_{nk})$, and $\boldsymbol{v}^{\otimes 2}=\boldsymbol{v}\boldsymbol{v}'$. A proof is provided in Supplementary Information Appendix A. On the other hand, for the multiplicative model $\mu _{ik}^M(\boldsymbol{Z}_{ik};\boldsymbol{\beta })$, the estimating equation for $\boldsymbol{\beta }$ can be simplified to

$$\begin{aligned} \sum ^K_{k=1}\sum ^n_{i=1}\left\{ \boldsymbol{Z}_{ik}-\bar{\boldsymbol{Z}}_k^M(\boldsymbol{\beta },\widehat{\boldsymbol{W}}_k)\right\} R_{ik}A_{ik}\widehat{W}_{ik}\varDelta ^Y_{ik}Y_{ik}=\boldsymbol{0}, \end{aligned}$$

where we set

$$\begin{aligned} \bar{\boldsymbol{Z}}_k^M(\boldsymbol{\beta },\boldsymbol{W}_k)= & \frac{\sum \limits ^{n}_{i=1}R_{ik}A_{ik}W_{ik}\varDelta ^Y_{ik}\exp (\boldsymbol{\beta }'\boldsymbol{Z}_{ik})\boldsymbol{Z}_{ik}}{\sum \limits ^{n}_{i=1}R_{ik}A_{ik}W_{ik}\varDelta ^Y_{ik}\exp (\boldsymbol{\beta }'\boldsymbol{Z}_{ik})}, \end{aligned}$$

and can be solved using iterative numerical techniques such as the Newton-Raphson method. A shortcut algorithm (Wang et al. 2019) using Cox regression software to obtain $\widehat{\boldsymbol{\beta }}$ can be applied in this case. The estimates for the landmark effect, $\widehat{\mu }_{0k}$, can then be obtained as a function of $\widehat{\boldsymbol{\beta }}$.

### Additional modeling considerations and extensions

First, we note that the above description of the estimation procedures implies that we stack the cross-section-specific data sets then (in the interests of parsimony) analyze the stacked data using a supermodel (instead of fitting landmark-specific models). An alternative would be to model cross-sections separately, perhaps after grouping in some fashion.

The parameter $\boldsymbol{\beta }$ in the stratified model represents the effect of $\boldsymbol{Z}_{ik}$ averaging over the time period from the *k*th cross-section up to the restriction time *L*. Therefore, the stratified model essentially assumes that the effect of $\boldsymbol{Z}_{ik}$ is invariant over cross-sections. However, it sometimes might be of scientific interest to characterize how the association between the RRMST and time-dependent covariates varies by cross-section, where time-varying coefficients have a well-defined clinical interpretation. In this case, the RRMST at the *k*th cross-section can be modeled by

7 $$\begin{aligned} \mu _{ik}(\boldsymbol{Z}_{ik};\boldsymbol{\beta }_k):=\mu _{0k}\exp \big (\boldsymbol{\beta }_k'\boldsymbol{Z}_{ik}\big ), \end{aligned}$$

where $\boldsymbol{\beta }_k$ can be a vector of *p* arbitrary parameters or a vector of *p* functions of *k*. If $\boldsymbol{\beta }_k$ is a vector of *p* arbitrary parameters, then model Eq. (7) is fully parametric with stratum-specific coefficients which allow for direct inference of the change in covariate effect from one landmark point to another. This model can be viewed as a fully stratified model (Zheng and Heagerty 2005) with $\boldsymbol{Z}_{ik}$ incorporating the interaction between the cross-sections and the pre-specified covariate functionals. Due to lack of parsimony in this model, when the number of cross-sections is large (*>100*), the model can be too dense to estimate. A potentially attractive alternative is to parametrize $\boldsymbol{\beta }_k$ in Eq. (7) as a smooth function of *k*. In addition to parsimony, such a specification enables extrapolation of risk assessment into future calendar period (i.e., beyond the calendar time period observed in the analysis).

The class of models as described by Eq. (7) is useful in visualization and evaluation of covariate effect trends over the chosen cross-sections. However, they are not helpful in building risk assessment tools because the covariate effects are assumed to be $\text {CS}_k$-dependent, i.e., calendar time-dependent. Instead of incorporating calendar time cross-sections in the risk model, follow-up times should be used as an alternative. Moreover, prior time survived is usually a strong indicator of a patient’s medical status and should be interpreted as a clinically meaningful quantity, and therefore it is reasonable to allow covariate effect at the *k*th cross-section to vary with $S_{ik}$ as follows:

8 $$\begin{aligned} \mu _{ik}\{\boldsymbol{Z}_{ik},S_{ik};\boldsymbol{\beta }(S_{ik})\}:=\mu _{0k}\exp \big \{\boldsymbol{\beta }(S_{ik})'\boldsymbol{Z}_{ik}\big \}, \end{aligned}$$

where $\boldsymbol{\beta }(S_{ik})$ is a vector of *p* functions of $S_{ik}$. Specification of $\boldsymbol{\beta }(S_{ik})$ usually follows scientific interpretation. For example, the effect of age may diminish as a patient lives long enough on the wait-list. A similar model (Yang et al. 2021) that has been used in the literature is $\mu _{ik}\{\boldsymbol{Z}_{ik},S_{ik};\boldsymbol{\beta }(S_{ik})\}=\boldsymbol{\beta }(S_{ik})'\boldsymbol{Z}_{ik}$.

Each of the above-mentioned models can be estimated using the proposed methods described in Sect. 2.4 with appropriate reparametrization and corresponding data augmentation. More discussion on the estimation procedure can be found in Supplementary Information Appendix B.

## Asymptotic properties

In this section, we first define some counting process-based quantities for statistical inference of the inverse probability weights. We then list the standard assumptions required to obtain the asymptotic normality of $\widehat{\boldsymbol{\beta }}$ and outline the asymptotic results.

Suppose $\boldsymbol{\theta }_{0C}$ is the unique solution to $\mathbb {E}\big [\int ^\tau _0\big \{\boldsymbol{Z}_i^C-\bar{\boldsymbol{Z}}^C(t;\boldsymbol{\theta }_C)\big \}dN_i^C(t)\big ]=\boldsymbol{0}$, and $\boldsymbol{\theta }_{0T}$ is the unique solution to $\mathbb {E}\big [\int ^\tau _0\big \{\boldsymbol{Z}_i^T(t)-\bar{\boldsymbol{Z}}^T(t;\boldsymbol{\theta }_T)\big \}dN_i^T(t)\big ]=\boldsymbol{0}$, where for patient *i,i=1,… ,n*, $N_i^C(t)=I(X_i\leqslant t,\varDelta ^D_i=0,\varDelta ^T_i=0)$ and $N_i^T(t)=I(X_i\leqslant t,\varDelta ^T_i=1)$ are the counting process of censoring and treatment times, respectively, $\bar{\boldsymbol{Z}}^C(t;\boldsymbol{\theta }_C)$ and $\bar{\boldsymbol{Z}}^T(t;\boldsymbol{\theta }_T)$ are defined in Web Appendix A. We define the following quantities based on the martingale process of censoring time, $M_i^C(t;\boldsymbol{\theta }_C)=N^C_i(t)-\int ^t_0R_i(u)d\varLambda ^C_i(u;\boldsymbol{\theta }_C)$, and the martingale process of treatment time, $M_i^T(t;\boldsymbol{\theta }_T)=N^T_i(t)-\int ^t_0A_i(u)d\varLambda ^T_i(u;\boldsymbol{\theta }_T)$.

$$\begin{aligned} \boldsymbol{U}^C_i(\boldsymbol{\theta }_C)&=\int ^\tau _0\big \{\boldsymbol{Z}^C_i-\bar{\boldsymbol{z}}^C(t;\boldsymbol{\theta }_C)\big \}dM^C_i(t;\boldsymbol{\theta }_C),\\ \boldsymbol{U}^T_i(\boldsymbol{\theta }_T)&=\int ^\tau _0\big \{\boldsymbol{Z}^T_i(t)-\bar{\boldsymbol{z}}^T(t;\boldsymbol{\theta }_T)\big \}dM^T_i(t;\boldsymbol{\theta }_T),\\ \boldsymbol{D}_{ik}^C(t;\boldsymbol{\theta }_C)&=\int ^{t+S_{ik}}_{S_{ik}}\big \{\boldsymbol{Z}^C_i-\bar{\boldsymbol{z}}^C(u;\boldsymbol{\theta }_C)\big \}d\varLambda ^C_i(u;\boldsymbol{\theta }_C),\\ \boldsymbol{k}^C_k(\boldsymbol{\beta },\boldsymbol{\theta }_C)&=\mathbb {E}\big \{R_{ik}A_{ik}\boldsymbol{\epsilon }_{ik}(\boldsymbol{\beta })\boldsymbol{D}_{ik}^C(Y_{ik};\boldsymbol{\theta }_C)'\big \},\\ \boldsymbol{\varOmega }^C(\boldsymbol{\theta }_C)&=\mathbb {E}\bigg [\int ^\tau _0\bigg \{\frac{\boldsymbol{s}^{(2)}_C(t;\boldsymbol{\theta }_C)}{s^{(0)}_C(t;\boldsymbol{\theta }_C)}-\bar{\boldsymbol{z}}^C(t;\boldsymbol{\theta }_C)^{\otimes 2}\bigg \}dM^C_i(t)\bigg ],\\ \boldsymbol{h}^C_k(t;\boldsymbol{\beta },\boldsymbol{\theta }_C)&=\mathbb {E}\big \{R_{ik}A_{ik}\boldsymbol{\epsilon }_{ik}(\boldsymbol{\beta })I(S_{ik}\leqslant t\leqslant S_{ik}+L)R_i(t)\exp \big (\boldsymbol{\theta }_C'\boldsymbol{Z}_i^C\big )\big \},\\ \boldsymbol{D}_{ik}^T(t;\boldsymbol{\theta }_T)&=\int ^{t+S_{ik}}_{S_{ik}}\big \{\boldsymbol{Z}^T_i(u)-\bar{\boldsymbol{z}}^T(u;\boldsymbol{\theta }_T)\big \}d\varLambda ^T_i(u;\boldsymbol{\theta }_T),\\ \boldsymbol{k}^T_k(\boldsymbol{\beta },\boldsymbol{\theta }_T)&=\mathbb {E}\big \{R_{ik}A_{ik}\boldsymbol{\epsilon }_{ik}(\boldsymbol{\beta })\boldsymbol{D}_{ik}^T(Y_{ik};\boldsymbol{\theta }_T)'\big \},\\ \boldsymbol{\varOmega }^T(\boldsymbol{\theta }_T)&=\mathbb {E}\bigg [\int ^\tau _0\bigg \{\frac{\boldsymbol{s}^{(2)}_T(t;\boldsymbol{\theta }_T)}{s^{(0)}_T(t;\boldsymbol{\theta }_T)}-\bar{\boldsymbol{z}}^T(t;\boldsymbol{\theta }_T)^{\otimes 2}\bigg \}dM^T_i(t)\bigg ],\\ \boldsymbol{h}^T_k(t;\boldsymbol{\beta },\boldsymbol{\theta }_T)&=\mathbb {E}\Big [R_{ik}A_{ik}\boldsymbol{\epsilon }_{ik}(\boldsymbol{\beta })I(S_{ik}\leqslant t\leqslant S_{ik}+L)R_i(t)\exp \big \{\boldsymbol{\theta }_T'\boldsymbol{Z}_i^T(t)\big \}\Big ], \end{aligned}$$

where $\bar{\boldsymbol{z}}^C(t;\boldsymbol{\theta }_C)$, $\bar{\boldsymbol{z}}^T(t;\boldsymbol{\theta }_T)$, $s^{(d)}_C(t;\boldsymbol{\theta }_C)$, $s^{(d)}_T(t;\boldsymbol{\theta }_T)$ are defined in the Supplementary Information Appendix A. We also assume the following regularity conditions hold for *i=1,… ,n* and *k=1,… ,K*.

(i) $\mathcal {O}_i=\big \{X_i,\varDelta ^D_i,\varDelta ^T_i,\widetilde{\boldsymbol{Z}}_i(X_i),\widetilde{A}_i(X_i)\big \}$ are independent and identically distributed random vectors.
(ii) $$P(X_i\geqslant t)>0$$ for $$t\in (0,\tau ]$$.
(iii) $\boldsymbol{Z}_i(t)$ is bounded almost surely.
(iv) $\varLambda _0^C(\tau )<\infty$, $\varLambda _0^T(\tau )<\infty$, and $\varLambda _0^C(t)$ and $\varLambda _0^T(t)$ are absolutely continuous for $t\in (0,\tau ]$, where $\varLambda _0^C(t)=\int ^t_0\lambda _0^C(u)du$ and $\varLambda _0^T(t)=\int ^t_0\lambda _0^T(u)du$.
(v) $\boldsymbol{A}^A(\boldsymbol{\beta })$, $\boldsymbol{A}^M(\boldsymbol{\beta })$, $\boldsymbol{\varOmega }^C(\boldsymbol{\theta }_C)$, $\boldsymbol{\varOmega }^T(\boldsymbol{\theta }_T)$ are positive definite, where
  $$\begin{aligned} \boldsymbol{A}^A(\boldsymbol{\beta })&=\mathbb {E}\bigg \{\sum \limits ^{K}_{k=1}R_{ik}A_{ik}W_{ik}\varDelta ^Y_{ik}(\boldsymbol{Z}_{ik}-\bar{\boldsymbol{z}}_k^A)^{\otimes 2}\bigg \},\\ \boldsymbol{A}^M(\boldsymbol{\beta })&=\mathbb {E}\bigg [\sum \limits ^{K}_{k=1}R_{ik}A_{ik}W_{ik}\varDelta ^Y_{ik}Y_{ik}\bigg \{\frac{\displaystyle s_k^{(2)}(\boldsymbol{\beta })}{\displaystyle s_k^{(0)}(\boldsymbol{\beta })}-\bar{\boldsymbol{z}}_k^M(\boldsymbol{\beta })^{\otimes 2}\bigg \}\bigg ], \end{aligned}$$
  with $\bar{\boldsymbol{z}}_k^A$, $s_k^{(d)}(\boldsymbol{\beta })$, and $\bar{\boldsymbol{z}}_k^M(\boldsymbol{\beta })$ defined in the Supplementary Information Appendix A.

### Theorem 1

Suppose $\boldsymbol{\beta }_0$ is the true value of $\boldsymbol{\beta }$, and $\widehat{\boldsymbol{\beta }}$ is the unique solution to the estimating Eqs. (5) and (6). Under the afore-listed regularity conditions, as $n\rightarrow \infty$, $\widehat{\boldsymbol{\beta }}$ converges in probability to $\boldsymbol{\beta }_0$ and $\sqrt{n}\big (\widehat{\boldsymbol{\beta }}-\boldsymbol{\beta }_0\big )$ converges to a zero-mean Normal distribution with variance $\boldsymbol{A}(\boldsymbol{\beta }_0)^{-1}\boldsymbol{B}(\boldsymbol{\beta }_0)\left\{ \boldsymbol{A}(\boldsymbol{\beta }_0)^{-1}\right\} '$, where $\boldsymbol{A}(\boldsymbol{\beta })=\boldsymbol{A}^A(\boldsymbol{\beta })$ for the additive model (1) and $\boldsymbol{A}(\boldsymbol{\beta })=\boldsymbol{A}^M(\boldsymbol{\beta })$ for the multiplicative model Eq. (2), respectively, and

$$\begin{aligned}&\boldsymbol{B}(\boldsymbol{\beta })=\mathbb {E}\bigg [\sum ^{K}_{k=1}\bigg \{R_{ik}A_{ik}\boldsymbol{\epsilon }_{ik}(\boldsymbol{\beta })\\&+\boldsymbol{k}^T_k(\boldsymbol{\beta },\boldsymbol{\theta }_{0T})\boldsymbol{\varOmega }^T(\boldsymbol{\theta }_{0T})^{-1}\boldsymbol{U}^T_i(\boldsymbol{\theta }_{0T})+\int ^\tau _0\frac{\boldsymbol{h}^T_k(t;\boldsymbol{\beta },\boldsymbol{\theta }_{0T})dM^T_i(t)}{s^{(0)}_T(t;\boldsymbol{\theta }_{0T})}\\&+\boldsymbol{k}^C_k(\boldsymbol{\beta },\boldsymbol{\theta }_{0C})\boldsymbol{\varOmega }^C(\boldsymbol{\theta }_{0C})^{-1}\boldsymbol{U}^C_i(\boldsymbol{\theta }_{0C})+\int ^\tau _0\frac{\boldsymbol{h}^C_k(t;\boldsymbol{\beta },\boldsymbol{\theta }_{0C})dM^C_i(t)}{s^{(0)}_C(t;\boldsymbol{\theta }_{0C})}\bigg \}\bigg ]^{\otimes 2}, \end{aligned}$$

with

$$\begin{aligned} \boldsymbol{\epsilon }_{ik}(\boldsymbol{\beta })=\left\{ \boldsymbol{Z}_{ik}-\bar{\boldsymbol{Z}}_k(\boldsymbol{\beta },\boldsymbol{W}_k)\right\} W_{ik}\varDelta ^Y_{ik}\left\{ Y_{ik}-\mu _{ik}(\boldsymbol{Z}_{ik};\boldsymbol{\beta })\right\} , \end{aligned}$$

where $\bar{\boldsymbol{Z}}_k(\boldsymbol{\beta },\boldsymbol{W}_k) = \bar{\boldsymbol{Z}}_k^A(\boldsymbol{W}_k)$ for the additive model (1) and $\bar{\boldsymbol{Z}}_k(\boldsymbol{\beta },\boldsymbol{W}_k) = \bar{\boldsymbol{Z}}_k^M(\boldsymbol{\beta },\boldsymbol{W}_k)$ for the multiplicative model Eq. (2), respectively.

The proof of Theorem 1 is available in Web Appendix A. To avoid computational complexity of the asymptotic variance, we treat the inverse probability weights $W_{ik}$ as fixed and offset $\boldsymbol{B}(\boldsymbol{\beta })$ to a simplified version, where we only retain the error term so that $\widetilde{\boldsymbol{B}}(\boldsymbol{\beta })=\text {E}\Big [\Big \{\sum \limits ^{K}_{k=1}R_{ik}A_{ik}\boldsymbol{\epsilon }_{ik}(\boldsymbol{\beta },\widehat{\boldsymbol{W}})\Big \}^{\otimes 2}\Big ]$. Wang and Schaubel (2018) evaluated two analogous sandwich variance estimators in a related set-up and showed that the simplified variance estimator well-approximates the variance estimator which includes martingale residuals from the counting processes. We will use the simplified variance estimator in our simulation study and data analysis.

## Simulation study

We conducted a simulation study to evaluate the performance of our proposed methods. Following the design described in Zheng and Heagerty (2005) and Gong and Schaubel (2013), we generated data from a hazards model with time-varying covariates, where both general (nonproportional) and proportional hazards were considered.

The study population is cross-sectioned at calendar dates $\text {CS}_k=k\times m$ for *k=1,...,K*, where *m* is the constant interval between two consecutive cross-sections. Suppose subject *i* entered the study on calendar date $E_i$, where $E_i$ followed a uniform distribution on (0, *b*). The follow-up time at the *k*th cross-section is then $S_{ik}=km-E_i$. We created a binary baseline/time-fixed covariate $Z_{ia}$, taking values 0 and 1 with equal probabilities, and a time-varying covariate $\boldsymbol{Z}_{ib}$, which was longitudinally observed with value $Z_{ib}(S_{ik})$ at the *k*th cross-section. To generate the sequence of $\boldsymbol{Z}_{ib}=\big (Z_{ib}(S_{ik})\big )$, *k=1,… ,K*, we created a baseline measure $Z_{ib0}=\eta _i+\sum \limits ^{K}_{k=1}\log (V_{ik})/\gamma _2$, where $\eta _i\overset{\text {i.i.d.}}{\sim }\mathcal {N}(\mu ,\sigma ^2)$. Let $Z_{ib}(S_{ik})=Z_{ib0}-\log (V_{ik})/\gamma _2$. Different specifications for $V_{ik}$ and $V_{i0}$ were considered to create the general (i.e., nonproportional) or proportional hazards model for failure time, $D_i$. We describe these two failure models below.

- *“General hazards” death model* Let $V_{ik}$ follow a truncated normal distribution with mean 0 and standard deviation 1 on the interval *(0,∞ )* and $V_{i0}$ follow a truncated normal distribution with mean 0 and standard deviation 2 on the interval *(0,∞ )*. $D_i$ was generated from the hazards model $\lambda _i(t)=V_{i0}\lambda _0(t)\exp (\gamma _1Z_{ia}+\gamma _2Z_{ib0})$, where $\varLambda _0(t)=\int ^t_0\lambda _0(u)du=(t/a)^{1/\nu }$. We set *ν =0.3*.
- *“Proportional hazards” (PH) death model* Let $V_{ik}\overset{\text {i.i.d.}}{\sim }S_\rho (\phi ,1,0)$ and $V_{i0}\overset{\text {i.i.d.}}{\sim }S_\rho (\phi ,1,0)$, where $S_\rho (\phi ,1,0)$ is a positive and strictly stable distribution with *0<ρ <1*. The properties of positive stable distribution warrant the proportionality of the partly conditional hazards model. We set $\phi =\left\{ a\cos (\pi \rho /2)\right\} ^{1/\rho }$ so that the Laplace transformation of $V_{ik}$ is $\mathbb {E}\left( e^{-tV_{ik}}\right) =\exp \left( -at^\rho \right)$ for *k=0,1,… ,K*. Suppose that death time, $D_i$, was generated from the hazards model $\lambda _i(t)=V_{i0}^{1/\rho }\lambda _0(t)\exp (\gamma _1Z_{ia}+\gamma _2Z_{ib0})$. We chose $\varLambda _0(t)=\int ^t_0\lambda _0(u)du=(t/a)^{1/\rho ^2}$, and the partly conditional hazards model was then $\lambda _{ik}\{t\mid Z_{ia},Z_{ib}(S_{ik}),D_i>S_{ik}\}=a^{\rho }\exp \left\{ \rho ^2\gamma _1Z_{ia}+\rho ^2\gamma _2Z_{ib}(S_{ik})\right\}$ which features a constant hazard rate with respect to the baseline covariate $Z_{ia}$ and the time-dependent covariate $Z_{ib}(S_{ik})$ over time.

Censoring time, $C_i$, was generated from the proportional hazards model $\lambda ^C_i(t)=\lambda ^C_0(t)\exp (\theta _CZ_{ia})$. The treatment eligibility process is an irreversible two-state model with one transition from treatment-eligible to treatment-ineligible. The time of treatment ineligibility, $A_i$, was generated from the proportional hazards model $\lambda ^A_i(t)=\lambda ^A_0(t)\exp (cV_{i0})$. Treatment time, $T_i$, was generated from $\lambda ^T_i(t)=A_i(t)\lambda ^T_0(t)\exp \big \{\theta _{T1}Z_{ia}+\theta _{T2}I(t>A_i)\big \}$, where $A_i(t)=I(t<A_i)$. The treatment model is conditional on the time-dependent covariate $I(t>A_i)$, which we assume was known as part of the observation. The covariate $V_{i0}$, however, was not measured during data collection and should be treated as unknown in the landmark analysis. The existence of $V_{i0}$ in both the death model (explicitly) and the treatment model (indirectly via $A_i$) ensures that $D_i$ and $T_i$ are dependent even conditional on measured covariates.

We set *K=5* and *m=10* to define the cross-sections and set *b=50* to define the entry times so that the study population has the same staggered entry scheme as our application. The parameter values in the death model are (1) general hazards model: $a=1,\mu =36,\sigma =1,\gamma _1=-0.6,\gamma _2=-0.3$, and (2) proportional hazards model: $\rho =0.8,a=1,\mu =24,\sigma =1,\gamma _1=-0.6,\gamma _2=-0.3$. The common parameter values in the censoring model are $\theta _C=0.001,\lambda _0^A(t)=0.002,c=0.001$. The common parameter values in the treatment model are $\theta _{T1}=-0.1,\theta _{T2}=0.1$. We vary the value of $\lambda _0^C(t)$ and $\lambda _0^T(t)$ to obtain different censoring probabilities. We consider two censoring patterns:

- *“Low” censoring* Approximately 15% of the sample population received treatment and 10% were censored. For the general hazards death model, we set $\lambda _0^C(t)=0.0014$ and $\lambda _0^T(t)=0.0028$. For the proportional hazards death model, we set $\lambda _0^C(t)=0.0012$ and $\lambda _0^T(t)=0.0024$.
- *“High” censoring* Approximately 30% of the sample population received treatment and 20% were censored. For the general hazards death model, we set $\lambda _0^C(t)=0.0040$ and $\lambda _0^T(t)=0.0075$. For the proportional hazards death model, we set $\lambda _0^C(t)=0.0040$ and $\lambda _0^T(t)=0.0077$.

For each scenario of censoring probability and death model, we consider the following working models for the landmark analysis: the additive model, $\mu _{ik}=\mu _{0k}+\beta _aZ_{ia}+\beta _bZ_{ikb}$, and the multiplicative model, $\mu _{ik}=\mu _{0k}\exp (\beta _aZ_{ia}+\beta _bZ_{ikb})$. The true parameter values were determined empirically using 10 million samples. We chose the restriction time to be *L=50* which approximated median of the death times $D_i$. For each scenario, we generated 1000 replications of *n=2500* samples to evaluate the bias, empirical standard deviation (ESD), average asymptotic standard error (ASE), and coverage probability (CP) of the estimators in each of the two models.

**Table 1:** Bias, empirical standard deviation (ESD), average asymptotic standard error (ASE), and coverage probability (CP) of the proposed estimators using the additive model for *L=50* and *K=5*, based on 1000 replications of *n=2500* samples

Censoring	Hazards	Parameter	Bias	ESD	ASE	CP (%)
Low	General	$\beta _a=2.75$	0.030	0.552	0.541	94.4
		$\beta _b=1.21$	0.001	0.034	0.034	95.9
	PH	$\beta _a=3.00$	−0.022	0.601	0.614	94.4
		$\beta _b=1.75$	0.004	0.048	0.050	95.2
High	General	$\beta _a=2.75$	−0.015	0.616	0.628	95.2
		$\beta _b=1.21$	−0.002	0.042	0.041	93.9
	PH	$\beta _a=3.00$	0.017	0.713	0.724	95.2
		$\beta _b=1.75$	0.021	0.062	0.057	92.2

Table 1 shows the analysis results applying the additive model. When a total censoring probability of 25% is considered, the model generates estimators with low bias under both general and proportional hazards generative mechanisms for death times. The ASE closely approximates the ESD, and the coverage probability differs only slightly from the nominal 95%. When the censoring probabilities increase to a total of 50%, the coefficient estimates associated with the baseline covariate ($\widehat{\beta }_a$) manage to maintain low bias and a reasonable coverage probability which is close to the nominal 95%. However, the results demonstrate a weaker coverage capability and non-negligible bias for the coefficient estimates associated with the time-dependent covariate ($\widehat{\beta }_b$) under the proportional hazards assumption, whereas $\widehat{\beta }_b$ under the general hazards mechanism remains to have low bias and a reasonable coverage probability. This disparity in the performance of the working model between general death hazards and proportional death hazards might be due to the heavier tail of the right-skewed stable distribution than that of the truncated normal distribution. In the presence of high censoring, the median of the observation times under proportional death hazards becomes much lower because the heavy tail is mostly censored. Choosing a restriction time that is much higher than the median of the observation times would result in some loss of power in detecting the true model parameters.

**Table 2:** Bias, empirical standard deviation (ESD), average asymptotic standard error (ASE), and coverage probability (CP) of the proposed estimators using the multiplicative model for *L=50* and *K=5*, based on 1000 replications of *n=2500* samples

Censoring	Hazards	Parameter	Bias	ESD	ASE	CP (%)
Low	General	$\beta _a=0.08$	−0.001	0.015	0.016	95.7
		$\beta _b=0.03$	0.000	0.001	0.001	95.0
	PH	$\beta _a=0.08$	0.001	0.016	0.016	95.2
		$\beta _b=0.05$	0.000	0.002	0.002	94.1
High	General	$\beta _a=0.08$	−0.001	0.018	0.018	94.6
		$\beta _b=0.03$	0.000	0.001	0.001	93.8
	PH	$\beta _a=0.08$	0.001	0.019	0.020	96.2
		$\beta _b=0.05$	0.001	0.003	0.002	90.2

Table 2 shows the analysis results applying the multiplicative model and demonstrates the same observation. The multiplicative model seems less robust in handling the heavy tail in the presence of high censoring and time-dependent covariates. Note that the proportional hazards setting is deliberately created for illustrative purposes, and it has been criticized for the impracticality to assume constant hazard ratio when covariates vary over time.

Simulation results described thus far have focused on absolute bias. In addition, we tabulate relative bias in the Supplementary Information (Appendix C.2, Table C.3). Relative biases for the additive and multiplicative models are comparable, and negligible in both cases.

We increased the number of cross-sections to *K=10* to test the estimation accuracy of our proposed working models. The simulation results are shown in the Supplementary Information Appendix C.1. In addition, we assessed the discrimination ability of our models. The concordance index ranged between 0.73 and 0.77 across all scenarios examined, with the additive and multiplicative models yielding similar concordance indices.

## Data analysis

We applied our proposed methods to liver transplant data obtained from the United Network for Organ Sharing (UNOS). The study population consists of *n=113,186* adult patients with ESLD who registered on the waiting list for a deceased donor liver between 1/1/2010 and 6/30/2021. Patient demographics and lab test results were recorded at the date of wait-listing, and a history of time-varying lab test results were collected longitudinally. The database was locked as of 6/30/2021, by which time 59% of wait-listed patients received a liver transplant, 20% died and 21% were censored.

Our primary analytic goal was to quantify, in terms of RRMST, the effect of risk factors (patient characteristics and longitudinally collected biomarkers) on survival in the absence of liver transplantation. We set landmark dates at the last day of each month from calendar years 2010 to 2019, resulting in a total of *K=120* cross-sections, and at each of these cross-sections we considered *L=3* months. That is, we assessed survival probability within an additional three-month window for patients who were at risk and treatment-eligible.

We adjusted for a comprehensive set of baseline and time-varying covariates. Baseline factors included age, sex, race, diabetes, liver disease diagnosis, malignancy, height, weight, functional status, and whether the patient was working for income. Time-varying predictors included dialysis status, ascites, encephalopathy, albumin, bilirubin (log-transformed), creatinine (capped at 4 and log-transformed), international normalized ratio (INR; log-transformed), and sodium. We also included the follow-up time at each landmark in the death model as a time-varying covariate.

The treatment model (used in building the inverse weight) included a similar set of covariates, but replacing the combination of bilirubin, creatinine, and INR in the death model with an allocation MELD score. The censoring model was adjusted for baseline demographics, the baseline values of time-varying covariates, and the date of wait-listing as an additional predictor. A summary of parameter estimates for the treatment model is available in the Supplementary Information Appendix D.1.

**Fig. 2:**
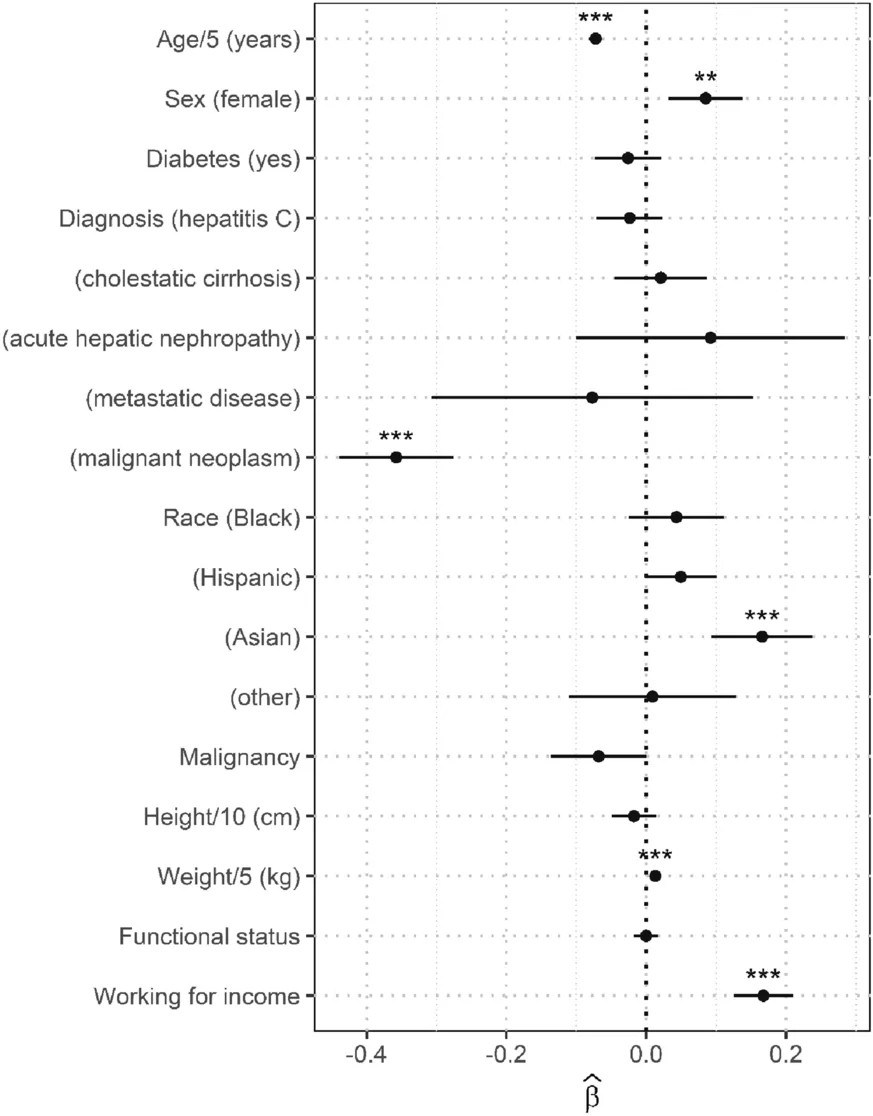
Effect estimates ($\widehat{\beta }_a$ in *days*) for baseline covariates and the corresponding 95% confidence interval using the additive model and monthly landmarking. Significance: * *p<0.05*, ** *p<0.01*, *** *p<0.001*

We fitted the additive model,

$$\begin{aligned} \mu _{ik}=\mu _{0k}+\sum ^{17}_{a=1}\beta _aZ_{ia}+\sum ^{9}_{b=1}\beta _bZ_{ikb}, \end{aligned}$$

where *k=1,… ,120*, $Z_{ia}$ represents baseline covariates, and $Z_{ikb}$ represents time-varying covariates. Figure 2 shows the effect estimates for the baseline covariates, the corresponding 95% confidence interval, and statistical significance of testing against $H_0:\widehat{\beta }_a=0$. Significant baseline covariates include age, sex, liver disease diagnosis (malignant neoplasm as compared to non-cholestatic cirrhosis), race (Asian as compared to White), weight, and whether the patient was working for income. Table 3 summarizes the effect estimates for the time-varying covariates, the corresponding standard error and *p*-value for testing against $H_0:\widehat{\beta }_b=0$. The significant time-varying covariates are albumin, bilirubin, creatinine, INR, serum sodium, and follow-up time. The direction of each of the covariate effects makes sense scientifically. Albumin stabilizes extracellular fluids and helps vitamins and hormones circulate in the body. A liver is functioning better if it synthesizes more albumin. Reduction in serum sodium is usually associated with hyponatremia in cirrhosis. Therefore, a higher level of albumin and serum sodium implies better liver condition and longer survival. On the other hand, high bilirubin implies severe liver disease, and high creatinine (chemical waste) implies impaired renal function, and hence higher risk of death is expected with increased level of bilirubin and creatinine. INR measures how long it takes for blood to clot, and a higher INR value indicates poor liver function. Therefore, the effect of INR on the remaining survival is expected to be negative. All the covariate effects are low in magnitude because only a very small proportion of patients died within the 3-month window; consequently, the stratum-specific intercepts are very close to 90 days, and the coefficients are correspondingly small. The low death rate reflects that the liver allocation system has largely succeeded in achieving its goal of minimizing deaths among wait-listed patients. We assessed the risk discrimination properties of the model using the concordance index with *m*-fold cross validation. In each fold, one of the *m* randomly partitioned groups of the original data was used as the test set and the remaining groups as the training set. For each fold, we constructed the stacked training and test data sets by pooling the cross-sectional data across landmarks, estimated the model parameters using the stacked training set, and computed a single concordance index by summing the numbers of concordant pairs and comparable pairs, respectively, across all cross-sections in the stacked test set. Note that we chose Harrell’s method to compute C-index for computational efficiency, as previous research (Gerds et al. 2013; Kim et al. 2018) has demonstrated that Harrell’s method, which does not explicitly model the censoring mechanism, performs as well as IPCW-based C-index estimators. Finally, we averaged the concordance indices across the *m* folds to obtain a reliable estimate of model discriminability. The concordance index of this predictive model, estimated with two-fold cross validation, is 0.698, indicating that the model has adequate discriminative ability. Increasing the number of folds yields a similar C-index, as the large sample size of the transplant registry database ensures stability and precision of our discrimination assessment. Note that the choice of *L* is important in defining the risk model as the parameter estimates change accordingly when different time windows are considered (Supplementary Information Appendix D.2). As the restriction window increases, biomarkers which are strong predictors of short-term survival may no longer be significant.

**Table 3:** Effect estimates ($\widehat{\beta }_b$ in *days*) for time-varying covariates with the corresponding standard error and *p*-value using the additive model and monthly landmarking

	$\widehat{\beta }_b$	se($\widehat{\beta }_b$)	*p*-value
Dialysis (Yes)	−0.09	0.130	0.46
Ascites	0.02	0.021	0.26
Encephalopathy	−0.01	0.022	0.64
Albumin	0.23	0.022	*<0.0001*
Bilirubin	−0.09	0.018	*<0.0001*
Creatinine	−0.37	0.081	*<0.0001*
INR	−0.36	0.081	*<0.0001*
Serum sodium	0.02	0.004	*<0.0001*
Follow-up time	−0.0001	0.00001	*<0.0001*

We also conducted the analysis using a variety of grids of landmark dates, the results of which are shown in the Supplementary Information Appendix D.3. If we set landmark dates at the end of each week starting from 1/1/2010 to 12/20/2019, then we have *K=520* cross-sections, and the results are quite similar to those based on monthly cross-sections. If we set landmark dates with longer intervals, such as landmarking at the end of each year (*K=10*), or half year (*K=20*) from 2010 to 2019, then we lose significance for some covariate effects (e.g., sex). We are able to identify the majority of the significant covariates with fewer cross-sections because the sample size of the liver transplant data is considerably large. We may not have this privilege of an extensive national registry data set in other applications, which further stresses the importance of using the stratified model to save the computation time required for setting a finer grid of landmark dates. The concordance index increases progressively with the number of cross-sections; however, the magnitude of this increase remains modest due to the large sample size of our registry data.

## Discussion

We proposed a novel approach to build risk models which facilitate dynamic updates and assessments of failure risk for individuals based on their emerging characteristics. Our proposed framework re-defined the endpoint using the RMST and generalized the context of time-to-event data to dependent censoring mechanism. The proposed methods would have a wide application in various medical fields where prognostic assessments are in great need. We carefully considered semiparametric direct RMST models for specifying covariate effects which are transferable to risk assessment for a future study. Note that these models are developed for dynamic risk assessment, whereas dynamic prediction models may need to take into account functions of landmark dates and prior time survived.

The proposed methods target residual survival time in the absence of treatment. Under this framework, the receipt of treatment censors (dependently, in our case) the absence-of-treatment survival time. This framework has a long history in survival analysis, dating back at least to Crowley and Hu (1977) and in other seminal works (Robins and Rotnitzky 1992; Robins and Finkelstein 2000). Another framework would view treatment, not as dependent censoring, but as a competing risk. For example, one could model the survival time lost due to death-without-treatment (i.e., *L-D*) in the setting where treatment time, *T*, is viewed as a competing risk that may or may not occur. Key methodology along these lines has been developed by Andersen (2013) and Conner and Trinquart (2021). The survival time lost in the competing risks setting is fundamentally different from the absence-of-treatment survival we consider. Rather than ruling out treatment receipt, the competing risks quantity implicitly averages over the occurrence/non-occurrence of the competing risk, treatment. Hence, the covariate effects on *(L-D)* are affected by treatment probability. In terms of transportability, two populations with the same covariate effects on absence-of-treatment survival, where *T* is perceived as dependent censoring, will generally have different effects on *(L-D)* unless the distributions of treatment times in the two studies are equal. The appeal of the competing risks perspective, as applied to the data structure in this report, is that it has a real-world-data interpretation. In the end, both the dependent censoring and competing risks perspectives have their pros and cons. The choice between the two should be driven by the scientific objectives. The dependent censoring perspective allows one to answer patient questions such as, “If I don’t get a liver transplant, what is my expected future survival time?” The competing risks framework addresses patient questions such as, “What is my expected time lost due to death-without-transplant, knowing that I may or may not get transplanted?”

The proposed methods require that treatment time be conditionally independent of survival in-the-absence-of-treatment given the observed longitudinal predictors. We formalize this assumption in Sect. 2.1. This assumption is analogous to the coarsening-at-random assumption underlying IPCW estimators generally (van der Laan and Robins 2003). The validity of this assumption (which is not testable) will depend on the application at hand. It could be argued that the assumption is approximately correct in our real-data example. Deceased-donor liver transplantation in the U.S. depends mostly on MELD score (as mandated) and region (in particular, donation service area). As reported by Sharma et al. (2012), patient characteristics do play a role and, as such, have been incorporated into our time-until-transplant model. Although the UNOS database does not contain every variable underlying LT probability, the most important LT predictors are available.

The proposed methods condition on treatment eligibility, *A(t)=1*. This choice is aligned with our objectives. In particular, it could be argued that patient counseling on absence-of-treatment prognosis is most relevant for patients who are candidates for receiving treatment. However, the appeal of this conditioning depends on the specifics of the data structure and analytic goals at hand. For cases where one does not wish to condition on *A(t)=1*, the proposed methods could still be applied by removing the conditionality from the main RRMST model and removing $A_{ik}$ from the estimating equations.

Implicitly, it is assumed that there is no feedback between the timing of the longitudinal measures and the failure time. Stated or not, this type of assumption underlies most methodologies in survival analysis dealing with time-dependent predictors. Although reasonable in many applied settings, this assumption may not hold in our liver transplant example. In particular, since increasing (decreasing) MELD score generally moves a patient up (down) the wait list, clinicians have a built-in incentive to obtain lab measurements when they suspect that their patient’s MELD score has increased (in which case, they are more likely to die). This appears to be a challenging issue to tackle, and one which to our knowledge has not been addressed in pertinent analysis of liver transplant candidates.

A common question in studying the RMST is how to select an optimal restriction time. This choice involves balancing clinical interpretation with statistical considerations. Clinically, there is often a conventional *L*-year survival time that investigators prioritize within their specific medical fields based on years of practice. Statistically, the restriction time should be large enough to make sure that a sufficient number of events have occurred in order to draw robust inference.

One of the less discussed limitations of directly modeling the RMST is that no survival function is emitted. Therefore, we used working models to describe the relations between the RMST and baseline/time-varying covariates. The models used in landmark analysis are working models. Although landmark model parameters are intended to provide useful information on the failure times of interest, the models are not intended to represent the data generating mechanism. This idea has been discussed in the literature for some time. Moreover, RMST models have long been considered to be working models, evidenced most explicitly by the fact that the restriction time is selected by the practitioner. Considerations such as these are referred to in comparing landmark analysis and its chief competitor, joint modeling. In making any comparisons between landmark analysis and joint modeling, at least four considerations are important. First, unlike landmark models, joint models can indeed be considered generative. Second, this perceived benefit of joint modeling is perhaps oversold since, for instance, any random effect parameters are assumed to follow a distribution that is numerically convenient as opposed to correct. Third, joint models are generally numerically cumbersome when multiple (even two) longitudinal variables are present. Fourth, most if not all joint models are based on the hazard function, which requires the assumed model to be correct at all points along the follow-up time continuum. In contrast, the RMST aims to have the model apply to a single time interval, rather than every point along that interval.

Use of LOCF in landmark analysis, where a missing measurement at the exact landmark date is imputed by the patient’s last observed value, is often criticized in literature for the staleness of the predictors which would lead to violations of the proportional hazards assumption (Putter and van Houwelingen 2022). While one of the advantages of directly modeling the RMST is indeed its robustness to such violations, the possible bias when there is a huge mismatch between the true biomarker and the imputed value has not been thoroughly studied and evaluated. A future direction to improve our proposed methods could focus on the two-stage approach (Huang et al. 2016; Rizopoulos et al. 2017) which bridges the LM and JM approaches and avoids using LOCF.

Another concern regarding the proposed additive model is the potential for the average residual RMST at a specific landmark to be negative. While this poses a challenge in risk prediction, where the ultimate goal is to obtain a predicted value for survival without treatment, it is less relevant in risk assessment. In the latter case, the focus is solely on the risk score, which is typically a function (e.g. linear combination) of patient characteristics and can be negative. This consideration also underscores the importance of stratifying the landmark intercepts. If survival prediction is of primary interest, then the multiplicative model is more suitable, where the exponential part of the model ensures that the stratification intercept remain nonnegative. However, since the focus of our research is risk score prediction, where a linear combination of predicting biomarkers may better reflect the reality, the multiplicative model may be less interpretable than the additive model.

## Supplementary Information

Below is the link to the electronic supplementary material.

Supplementary file 1 (pdf 265 KB)

## Data Availability

The programming scripts for simulation and data analysis, in the form of R code, are available on https://github.com/YZhang469/UPenn-RRMSTLM.
