# Supplementary material for: Dynamic risk assessment by landmark modeling of the restricted residual mean survival time
Source: Lifetime Data Anal. 2026 Oct 20;32(4):56. doi: 10.1007/s10985-026-09731-z (PMC13683509; doi:10.1007/s10985-026-09731-z)
Supplement: Supplementary file 1 [file 10985_2026_9731_MOESM1_ESM.pdf]

Supplementary Information for "Dynamic Risk Assessment by Landmark Modeling of the Restricted Residual Mean Survival Time"

Appendix A Asymptotic Properties of the Proposed Estimator

A.1 Summary of Notation and Expressions

For subject i, where $i = 1, \ldots, n$,

- $R_i(t) = I(X_i \geqslant t)$: at-risk indicator
- $A_i(t) \in \{0, 1\}$: time-varying treatment eligibility
- $\widetilde{A}_i(t) = \{A_i(s); s \in [0, t]\}$: treatment-eligibility history
- D_i: survival time, which may be censored at treatment time T_i or administrative censoring time C_i
- $X_i = D_i \wedge T_i \wedge C_i$: observed treatment-free event time
- $\Delta_i^D = I(X_i = D_i) = I(D_i < T_i \wedge C_i)$
- $\Delta_i^T = I(X_i = T_i) = I(T_i < D_i \wedge C_i)$
- $\boldsymbol{Z}_i(t)$: time-varying covariates
- $\widetilde{\boldsymbol{Z}}_i(t) = \{\boldsymbol{Z}_i(s); s \in [0, t]\}$: covariate history
- CS_k, where $k = 1, \ldots, K$: calendar date of the kth cross-section
- S_{ik}: follow-up time until CS_k
- $R_{ik} = R_i(S_{ik})$: at-risk indicator at the kth cross-section
- $A_{ik} = A_i(S_{ik})$: treatment eligibility at the kth cross-section
- $D_{ik} = D_i - S_{ik}$: the remaining death time at the kth cross-section
- $T_{ik} = T_i - S_{ik}$: the remaining treatment time at the kth cross-section
- $C_{ik} = C_i - S_{ik}$: the remaining censoring time at the kth cross-section
- $X_{ik} = D_{ik} \wedge T_{ik} \wedge C_{ik} = X_i - S_{ik}$: the remaining observed time at the kth cross-section
- $\Delta_{ik}^D = I(X_{ik} = D_{ik}) = I(D_{ik} < T_{ik} \wedge C_{ik})$
- $\Delta_{ik}^T = I(X_{ik} = T_{ik}) = I(T_{ik} < D_{ik} \wedge C_{ik})$
- $Y_{ik} = X_{ik} \wedge L$: treatment-free survival time up to L at the kth cross-section

- $\Delta_{ik}^{Y} = I(Y_{ik} = D_{ik} \wedge L) = I(D_{ik} \wedge L < T_{ik} \wedge C_{ik})$
- $\boldsymbol{Z}_{ik} = \boldsymbol{Z}_i(S_{ik})$: covariates measured at the kth cross-section
- $\mu_{ik}(\boldsymbol{Z}_{ik};\boldsymbol{\beta}) := \mathbb{E}\big(D_{ik} \wedge L | \boldsymbol{Z}_{ik}, A_{ik} = 1\big)$: user-specified model of the restricted mean survival time at the kth cross-section; $\mu_{ik}(\boldsymbol{Z}_{ik};\boldsymbol{\beta})$ can take the form of $\mu_{0k} + \boldsymbol{\beta}'\boldsymbol{Z}_{ik}$ or $\mu_{0k}\exp(\boldsymbol{\beta}'\boldsymbol{Z}_{ik})$
- Cox proportional hazards model of censoring times: $\lambda_i^C(t) = R_i(t)\lambda_0^C(t)\exp\left\{\boldsymbol{\theta}_C'\boldsymbol{Z}_i^C\right\}$
- Inverse probability of censoring weights (IPCWs): $W_{ik}^C = \exp\left\{\Lambda_i^C(Y_{ik} + S_{ik}) - \Lambda_i^C(S_{ik})\right\}$, where $\Lambda_i^C(t) = \int_0^t \lambda_i^C(u)du$
- Cox proportional hazards model of treatment times: $\lambda_i^T(t) = A_i(t)\lambda_0^T(t)\exp\left\{\boldsymbol{\theta}_T'\boldsymbol{Z}_i^T(t)\right\}$
- Inverse probability of treatment weights (IPTWs): $W_{ik}^T = \exp\left\{\Lambda_i^T(Y_{ik} + S_{ik}) - \Lambda_i^T(S_{ik})\right\}$, where $\Lambda_i^T(t) = \int_0^t \lambda_i^T(u)du$
- Expressions in derivation of asymptotic normality:

$$\bar{\boldsymbol{Z}}_k^A(\boldsymbol{W}_k) = \frac{\sum_{i=1}^n \boldsymbol{Z}_{ik}R_{ik}A_{ik}W_{ik}\Delta_{ik}^Y}{\sum_{i=1}^n R_{ik}A_{ik}W_{ik}\Delta_{ik}^Y} \qquad \bar{\boldsymbol{z}}_k^A = \frac{\mathbb{E}\left(\boldsymbol{Z}_{ik}R_{ik}A_{ik}\right)}{\mathbb{E}\left(R_{ik}A_{ik}\right)}$$

$$\boldsymbol{S}_k^{(d)}(\boldsymbol{\beta}, \boldsymbol{W}_k) = \frac{1}{n}\sum_{i=1}^n \boldsymbol{Z}_{ik}^{\otimes d}R_{ik}A_{ik}W_{ik}\Delta_{ik}^Y\exp\left(\boldsymbol{\beta}'\boldsymbol{Z}_{ik}\right) \qquad \boldsymbol{s}_k^{(d)}(\boldsymbol{\beta}) = \mathbb{E}\left\{\boldsymbol{Z}_{ik}^{\otimes d}R_{ik}A_{ik}\exp\left(\boldsymbol{\beta}'\boldsymbol{Z}_{ik}\right)\right\}$$

$$\bar{\boldsymbol{Z}}_k^M(\boldsymbol{\beta}, \boldsymbol{W}_k) = \frac{\boldsymbol{S}_k^{(1)}(\boldsymbol{\beta}, \boldsymbol{W}_k)}{S_k^{(0)}(\boldsymbol{\beta}, \boldsymbol{W}_k)} \qquad \bar{\boldsymbol{z}}_k^M(\boldsymbol{\beta}) = \frac{\boldsymbol{s}_k^{(1)}(\boldsymbol{\beta})}{s_k^{(0)}(\boldsymbol{\beta})}$$

$$\boldsymbol{S}_C^{(d)}(t;\boldsymbol{\theta}_C) = \frac{1}{n}\sum_{i=1}^n \boldsymbol{Z}_i^{C\otimes d}R_i(t)\exp\left(\boldsymbol{\theta}_C'\boldsymbol{Z}_i^C\right) \qquad \boldsymbol{s}_C^{(d)}(t;\boldsymbol{\theta}_C) = \mathbb{E}\left\{\boldsymbol{Z}_i^{C\otimes d}R_i(t)\exp\left(\boldsymbol{\theta}_C'\boldsymbol{Z}_i^C\right)\right\}$$

$$\bar{\boldsymbol{Z}}^C(t;\boldsymbol{\theta}_C) = \frac{\boldsymbol{S}_C^{(1)}(t;\boldsymbol{\theta}_C)}{S_C^{(0)}(t;\boldsymbol{\theta}_C)} \qquad \bar{\boldsymbol{z}}^C(t;\boldsymbol{\theta}_C) = \frac{\boldsymbol{s}_C^{(1)}(t;\boldsymbol{\theta}_C)}{s_C^{(0)}(t;\boldsymbol{\theta}_C)}$$

$$\boldsymbol{S}_T^{(d)}(t;\boldsymbol{\theta}_T) = \frac{1}{n}\sum_{i=1}^n \boldsymbol{Z}_i^T(t)^{\otimes d}R_i(t)\exp\left\{\boldsymbol{\theta}_T'\boldsymbol{Z}_i^T(t)\right\} \qquad \boldsymbol{s}_T^{(d)}(t;\boldsymbol{\theta}_T) = \mathbb{E}\left[\boldsymbol{Z}_i^T(t)^{\otimes d}R_i(t)\exp\left\{\boldsymbol{\theta}_T'\boldsymbol{Z}_i^T(t)\right\}\right]$$

$$\bar{\boldsymbol{Z}}^T(t;\boldsymbol{\theta}_T) = \frac{\boldsymbol{S}_T^{(1)}(t;\boldsymbol{\theta}_T)}{S_T^{(0)}(t;\boldsymbol{\theta}_T)} \qquad \bar{\boldsymbol{z}}^T(t;\boldsymbol{\theta}_T) = \frac{\boldsymbol{s}_T^{(1)}(t;\boldsymbol{\theta}_T)}{s_T^{(0)}(t;\boldsymbol{\theta}_T)}$$

where $\boldsymbol{v}^{\otimes 0} = 1$, $\boldsymbol{v}^{\otimes 1} = \boldsymbol{v}$, $\boldsymbol{v}^{\otimes 2} = \boldsymbol{v}\boldsymbol{v}'$, and $\boldsymbol{W}_k = (W_{1k}, \ldots, W_{nk})$.

A.2 Unbiasedness of Estimating Equations

The estimating equations for the landmark RMST model are

$$\sum_{k=1}^K\sum_{i=1}^n R_{ik}A_{ik}W_{ik}\Delta_{ik}^Y\boldsymbol{Z}_{ik}\left\{Y_{ik} - \mu_{ik}(\boldsymbol{Z}_{ik};\boldsymbol{\beta})\right\} = \boldsymbol{0}, \tag{1}$$

$$\sum_{i=1}^n R_{ik}A_{ik}W_{ik}\Delta_{ik}^Y\left\{Y_{ik} - \mu_{ik}(\boldsymbol{Z}_{ik};\boldsymbol{\beta})\right\} = 0, \qquad k = 1, \ldots, K. \tag{2}$$

Estimating equation (1) is unbiased since

$$\mathbb{E}\Big[R_{ik}A_{ik}W_{ik}\Delta_{ik}^Y\boldsymbol{Z}_{ik}\big\{Y_{ik} - \mu_{ik}(\boldsymbol{Z}_{ik};\boldsymbol{\beta})\big\}\Big]$$

$$
\begin{aligned}
&= \mathbb{E}\Big[I(X_i > S_{ik})A_{ik}\frac{I(D_{ik}\wedge L < T_{ik}\wedge C_{ik})}{P\big(C_{ik} > Y_{ik}\big|X_i > S_{ik}, X_{ik}, \boldsymbol{Z}_i^C\big)P\big\{T_{ik} > Y_{ik}\big|X_i > S_{ik}, X_{ik}, \boldsymbol{Z}_i^T(S_{ik})\big\}}\boldsymbol{Z}_{ik}\big\{Y_{ik} - \mu_{ik}(\boldsymbol{Z}_{ik};\boldsymbol{\beta})\big\}\Big] \\
&= \mathbb{E}\Big[I(D_i > S_{ik})A_{ik}\frac{I(D_{ik}\wedge L < T_{ik}\wedge C_{ik})\boldsymbol{Z}_{ik}\big\{D_{ik}\wedge L - \mu_{ik}(\boldsymbol{Z}_{ik};\boldsymbol{\beta})\big\}}{P\big(C_{ik} > D_{ik}\wedge L\big|D_i > S_{ik}, D_{ik}, \boldsymbol{Z}_i^C\big)P\big\{T_{ik} > D_{ik}\wedge L\big|D_i > S_{ik}, D_{ik}, \boldsymbol{Z}_i^T(S_{ik})\big\}}\Big] \\
&= P\big(D_i > S_{ik}, A_{ik} = 1\big)\mathbb{E}\Big[\frac{I(D_{ik}\wedge L < T_{ik}\wedge C_{ik})\boldsymbol{Z}_{ik}\big\{D_{ik}\wedge L - \mu_{ik}(\boldsymbol{Z}_{ik};\boldsymbol{\beta})\big\}}{P\big(C_{ik} > D_{ik}\wedge L\big|D_i > S_{ik}, D_{ik}, \boldsymbol{Z}_i^C\big)P\big\{T_{ik} > D_{ik}\wedge L\big|D_i > S_{ik}, D_{ik}, \boldsymbol{Z}_i^T(S_{ik})\big\}}\Big|D_i > S_{ik}, A_{ik} = 1\Big] \\
&= P\big(D_i > S_{ik}, A_{ik} = 1\big)\mathbb{E}\Big\{\mathbb{E}\Big[\frac{I(D_{ik}\wedge L < T_{ik}\wedge C_{ik})\boldsymbol{Z}_{ik}\big\{D_{ik}\wedge L - \mu_{ik}(\boldsymbol{Z}_{ik};\boldsymbol{\beta})\big\}}{P\big(C_{ik} > D_{ik}\wedge L\big|D_i > S_{ik}, D_{ik}, \boldsymbol{Z}_i^C\big)P\big\{T_{ik} > D_{ik}\wedge L\big|D_i > S_{ik}, D_{ik}, \boldsymbol{Z}_i^T(S_{ik})\big\}} \\
&\qquad\qquad \Big|D_i > S_{ik}, A_{ik} = 1, D_{ik}, \boldsymbol{Z}_{ik}, \boldsymbol{Z}_i^C, \boldsymbol{Z}_i^T(S_{ik})\Big]\Big|D_i > S_{ik}, A_{ik} = 1\Big\} \\
&= P\big(D_i > S_{ik}, A_{ik} = 1\big)\mathbb{E}\Big[\frac{P\big\{D_{ik}\wedge L < T_{ik}\wedge C_{ik}\big|D_i > S_{ik}, A_{ik} = 1, D_{ik}, \boldsymbol{Z}_{ik}, \boldsymbol{Z}_i^C, \boldsymbol{Z}_i^T(S_{ik})\big\}}{P\big(C_{ik} > D_{ik}\wedge L\big|D_i > S_{ik}, D_{ik}, \boldsymbol{Z}_i^C\big)P\big\{T_{ik} > D_{ik}\wedge L\big|D_i > S_{ik}, D_{ik}, \boldsymbol{Z}_i^T(S_{ik})\big\}} \\
&\qquad\qquad \boldsymbol{Z}_{ik}\big\{D_{ik}\wedge L - \mu_{ik}(\boldsymbol{Z}_{ik};\boldsymbol{\beta})\big\}\Big|D_i > S_{ik}, A_{ik} = 1\Big] \\
&= P\big(D_i > S_{ik}, A_{ik} = 1\big)\mathbb{E}\Big[\frac{P\big(D_{ik}\wedge L < C_{ik}\big|D_i > S_{ik}, D_{ik}, \boldsymbol{Z}_i^C\big)P\big\{D_{ik}\wedge L < T_{ik}\big|D_i > S_{ik}, D_{ik}, \boldsymbol{Z}_i^T(S_{ik})\big\}}{P\big(C_{ik} > D_{ik}\wedge L\big|D_i > S_{ik}, D_{ik}, \boldsymbol{Z}_i^C\big)P\big\{T_{ik} > D_{ik}\wedge L\big|D_i > S_{ik}, D_{ik}, \boldsymbol{Z}_i^T(S_{ik})\big\}} \\
&\qquad\qquad \boldsymbol{Z}_{ik}\big\{D_{ik}\wedge L - \mu_{ik}(\boldsymbol{Z}_{ik};\boldsymbol{\beta})\big\}\Big|D_i > S_{ik}, A_{ik} = 1\Big] \\
&= P\big(D_i > S_{ik}, A_{ik} = 1\big)\mathbb{E}\Big[\boldsymbol{Z}_{ik}\big\{D_{ik}\wedge L - \mu_{ik}(\boldsymbol{Z}_{ik};\boldsymbol{\beta})\big\}\Big|D_i > S_{ik}, A_{ik} = 1\Big] \\
&= P\big(D_i > S_{ik}, A_{ik} = 1\big)\mathbb{E}\Big\{\mathbb{E}\Big[\boldsymbol{Z}_{ik}\big\{D_{ik}\wedge L - \mu_{ik}(\boldsymbol{Z}_{ik};\boldsymbol{\beta})\big\}\Big|D_i > S_{ik}, A_{ik} = 1, \boldsymbol{Z}_{ik}\Big]\Big|D_i > S_{ik}, A_{ik} = 1\Big\} \\
&= P\big(D_i > S_{ik}, A_{ik} = 1\big)\mathbb{E}\Big[\boldsymbol{Z}_{ik}\Big\{\mathbb{E}\big(D_{ik}\wedge L\big|D_i > S_{ik}, A_{ik} = 1, \boldsymbol{Z}_{ik}\big) - \mu_{ik}(\boldsymbol{Z}_{ik};\boldsymbol{\beta})\Big\}\Big|D_i > S_{ik}, A_{ik} = 1\Big] \\
&= \boldsymbol{0},
\end{aligned}
$$

and the proof for the unbiasedness of estimating equation (2) follows analogously.

A.3 Closed-Form Expression for $\boldsymbol{\beta}$ in the Additive Model

For the additive model $\mu_{ik}(\boldsymbol{Z}_{ik};\boldsymbol{\beta}) = \mu_{0k} + \boldsymbol{\beta}'\boldsymbol{Z}_{ik}$, from equation (2),

$$
\mu_{0k} = \frac{\sum_{i=1}^{n} R_{ik}A_{ik}W_{ik}\Delta_{ik}^{Y}\left(Y_{ik} - \boldsymbol{\beta}'\boldsymbol{Z}_{ik}\right)}{\sum_{i=1}^{n} R_{ik}A_{ik}W_{ik}\Delta_{ik}^{Y}}.
$$

Let $\bar{\boldsymbol{Z}}_k(\boldsymbol{W}_k) = \dfrac{\sum_{i=1}^{n} R_{ik}A_{ik}W_{ik}\Delta_{ik}^{Y}\boldsymbol{Z}_{ik}}{\sum_{i=1}^{n} R_{ik}A_{ik}W_{ik}\Delta_{ik}^{Y}}$. Equation (1) can be written as

$$
\sum_{k=1}^{K}\sum_{i=1}^{n}\Big\{\boldsymbol{Z}_{ik} - \bar{\boldsymbol{Z}}_k(\boldsymbol{W}_k)\Big\}R_{ik}A_{ik}W_{ik}\Delta_{ik}^{Y}\left(Y_{ik} - \boldsymbol{\beta}'\boldsymbol{Z}_{ik}\right) = \boldsymbol{0}.
$$

It follows that

$$
\sum_{k=1}^{K}\sum_{i=1}^{n}\Big\{\boldsymbol{Z}_{ik} - \bar{\boldsymbol{Z}}_k(\boldsymbol{W}_k)\Big\}R_{ik}A_{ik}W_{ik}\Delta_{ik}^{Y}Y_{ik} = \Big[\sum_{k=1}^{K}\sum_{i=1}^{n}\Big\{\boldsymbol{Z}_{ik} - \bar{\boldsymbol{Z}}_k(\boldsymbol{W}_k)\Big\}\boldsymbol{Z}_{ik}'R_{ik}A_{ik}W_{ik}\Delta_{ik}^{Y}\Big]\boldsymbol{\beta}
$$

$$= \left[\sum_{k=1}^{K}\sum_{i=1}^{n}\Big\{\boldsymbol{Z}_{ik} - \bar{\boldsymbol{Z}}_k(\boldsymbol{W}_k)\Big\}\Big\{\boldsymbol{Z}_{ik} - \bar{\boldsymbol{Z}}_k(\boldsymbol{W}_k)\Big\}' R_{ik}A_{ik}W_{ik}\Delta_{ik}^{Y}\right]\boldsymbol{\beta}.$$

Thus,

$$\boldsymbol{\beta} = \left[\sum_{k=1}^{K}\sum_{i=1}^{n}\Big\{\boldsymbol{Z}_{ik} - \bar{\boldsymbol{Z}}_k(\boldsymbol{W}_k)\Big\}^{\otimes 2} R_{ik}A_{ik}W_{ik}\Delta_{ik}^{Y}\right]^{-1}\left[\sum_{k=1}^{K}\sum_{i=1}^{n}\Big\{\boldsymbol{Z}_{ik} - \bar{\boldsymbol{Z}}_k(\boldsymbol{W}_k)\Big\}R_{ik}A_{ik}W_{ik}\Delta_{ik}^{Y}Y_{ik}\right].$$

A.4 Regularity Conditions

Suppose $\boldsymbol{\beta}_0$ is the unique solution to $\sum_{k=1}^{K}\mathbb{E}\Big[R_{ik}A_{ik}W_{ik}\Delta_{ik}^{Y}\boldsymbol{Z}_{ik}\big\{Y_{ik} - \mu_{ik}(\boldsymbol{Z}_{ik};\boldsymbol{\beta})\big\}\Big] = \mathbf{0}$, $\boldsymbol{\theta}_{0C}$ is the unique solution to $\mathbb{E}\Big[\int_0^\tau \big\{\boldsymbol{Z}_i^C - \bar{\boldsymbol{Z}}^C(t;\boldsymbol{\theta}_C)\big\}\, dN_i^C(t)\Big] = \mathbf{0}$, and $\boldsymbol{\theta}_{0T}$ is the unique solution to $\mathbb{E}\Big[\int_0^\tau \big\{\boldsymbol{Z}_i^T(t) - \bar{\boldsymbol{Z}}^T(t;\boldsymbol{\theta}_T)\big\}\, dN_i^T(t)\Big] = \mathbf{0}$, where $N_i^C(t)$ and $N_i^T(t)$ are the counting process of censoring and treatment times, respectively, for patient $i, i = 1,\ldots,n$. We assume the following regularity conditions for $i = 1,\ldots,n$ and $k = 1,\ldots,K$:

(i) $\mathcal{O}_i = \big\{X_i, \Delta_i^D, \Delta_i^T, \widetilde{\boldsymbol{Z}}_i(X_i), \widetilde{A}_i(X_i)\big\}$ are independent and identically distributed random vectors.

(ii) $P(X_i \geqslant t) > 0$ for $t \in (0,\tau]$.

(iii) $\boldsymbol{Z}_i(t)$ is bounded almost surely.

(iv) $\Lambda_0^C(\tau) < \infty$, $\Lambda_0^T(\tau) < \infty$, and $\Lambda_0^C(t)$ and $\Lambda_0^T(t)$ are absolutely continuous for $t \in (0,\tau]$, where $\Lambda_0^C(t) = \int_0^t \lambda_0^C(u)du$ and $\Lambda_0^T(t) = \int_0^t \lambda_0^T(u)du$.

(v) $\boldsymbol{A}^A(\boldsymbol{\beta})$, $\boldsymbol{A}^M(\boldsymbol{\beta})$, $\boldsymbol{\Omega}^C(\boldsymbol{\theta}_C)$, $\boldsymbol{\Omega}^T(\boldsymbol{\theta}_T)$ are positive definite, where

$$\boldsymbol{A}^A(\boldsymbol{\beta}) = \mathbb{E}\bigg\{\sum_{k=1}^{K} R_{ik}A_{ik}W_{ik}\Delta_{ik}^{Y}(\boldsymbol{Z}_{ik} - \bar{\boldsymbol{z}}_k^{A})^{\otimes 2}\bigg\},$$

$$\boldsymbol{A}^M(\boldsymbol{\beta}) = \mathbb{E}\left[\sum_{k=1}^{K} R_{ik}A_{ik}W_{ik}\Delta_{ik}^{Y}Y_{ik}\left\{\frac{s_k^{(2)}(\boldsymbol{\beta})}{s_k^{(0)}(\boldsymbol{\beta})} - \bar{\boldsymbol{z}}_k^{M}(\boldsymbol{\beta})^{\otimes 2}\right\}\right],$$

$$\boldsymbol{\Omega}^C(\boldsymbol{\theta}_C) = \mathbb{E}\left[\int_0^\tau \left\{\frac{\boldsymbol{s}_C^{(2)}(t;\boldsymbol{\theta}_C)}{s_C^{(0)}(t;\boldsymbol{\theta}_C)} - \bar{\boldsymbol{z}}^C(t;\boldsymbol{\theta}_C)^{\otimes 2}\right\}dM_i^C(t)\right],$$

$$\boldsymbol{\Omega}^T(\boldsymbol{\theta}_T) = \mathbb{E}\left[\int_0^\tau \left\{\frac{\boldsymbol{s}_T^{(2)}(t;\boldsymbol{\theta}_T)}{s_T^{(0)}(t;\boldsymbol{\theta}_T)} - \bar{\boldsymbol{z}}^T(t;\boldsymbol{\theta}_T)^{\otimes 2}\right\}dM_i^T(t)\right].$$

Under regularity conditions,

$$\sqrt{n}\big(\widehat{\boldsymbol{\theta}}_C - \boldsymbol{\theta}_{0C}\big) = \frac{1}{\sqrt{n}}\left[\frac{1}{n}\sum_{i=1}^{n}\int_0^\tau \left\{\frac{\boldsymbol{s}_C^{(2)}(t;\boldsymbol{\theta}_{0C})}{s_C^{(0)}(t;\boldsymbol{\theta}_{0C})} - \bar{\boldsymbol{z}}^C(t;\boldsymbol{\theta}_{0C})^{\otimes 2}\right\}dM_i^C(t)\right]^{-1}\sum_{i=1}^{n}\boldsymbol{U}_i^C(\boldsymbol{\theta}_{0C}) + o_p(1),$$

$$\sqrt{n}\big(\widehat{\boldsymbol{\theta}}_T - \boldsymbol{\theta}_{0T}\big) = \frac{1}{\sqrt{n}}\left[\frac{1}{n}\sum_{i=1}^{n}\int_0^\tau \left\{\frac{\boldsymbol{s}_T^{(2)}(t;\boldsymbol{\theta}_{0T})}{s_T^{(0)}(t;\boldsymbol{\theta}_{0T})} - \bar{\boldsymbol{z}}^T(t;\boldsymbol{\theta}_{0T})^{\otimes 2}\right\}dM_i^T(t)\right]^{-1}\sum_{i=1}^{n}\boldsymbol{U}_i^T(\boldsymbol{\theta}_{0T}) + o_p(1),$$

where

$$\boldsymbol{U}_i^C(\boldsymbol{\theta}_C) = \int_0^\tau \big\{\boldsymbol{Z}_i^C - \bar{\boldsymbol{z}}^C(t;\boldsymbol{\theta}_C)\big\}\, dM_i^C(t),$$

$$\boldsymbol{U}_i^T(\boldsymbol{\theta}_T) = \int_0^\tau \left\{\boldsymbol{Z}_i^T(t) - \bar{\boldsymbol{z}}^T(t;\boldsymbol{\theta}_T)\right\} dM_i^T(t).$$

Following the derivation in **?**, we first note that for the centered process of the estimated cumulative hazards of censoring can be written as

$$\sqrt{n}\left\{\widehat{\Lambda}_i^C(t;\widehat{\boldsymbol{\theta}}_C) - \Lambda_i^C(t;\boldsymbol{\theta}_{0C})\right\} = \sqrt{n}\left\{\widehat{\Lambda}_i^C(t;\widehat{\boldsymbol{\theta}}_C) - \widehat{\Lambda}_i^C(t;\boldsymbol{\theta}_{0C})\right\} \tag{3}$$

$$+ \sqrt{n}\left\{\widehat{\Lambda}_i^C(t;\boldsymbol{\theta}_{0C}) - \Lambda_i^C(t;\boldsymbol{\theta}_{0C})\right\}. \tag{4}$$

Using the Taylor expansion of $\widehat{\Lambda}_i^C(t;\widehat{\boldsymbol{\theta}}_C)$ at $\boldsymbol{\theta}_{0C}$,

$$\begin{aligned}
(3) &= \frac{\partial}{\partial \boldsymbol{\theta}_{0C}'}\widehat{\Lambda}_i^C(t;\boldsymbol{\theta}_{0C}) \cdot \sqrt{n}\big(\widehat{\boldsymbol{\theta}}_C - \boldsymbol{\theta}_{0C}\big) + o_p(1) \\
&= \int_0^t \left\{\boldsymbol{Z}_i^C - \bar{\boldsymbol{z}}^C(u;\boldsymbol{\theta}_{0C})\right\}' d\Lambda_i^C(u;\boldsymbol{\theta}_{0C}) \cdot \sqrt{n}\big(\widehat{\boldsymbol{\theta}}_C - \boldsymbol{\theta}_{0C}\big) + o_p(1) \\
&= \frac{1}{\sqrt{n}}\boldsymbol{D}_i^C(t;\boldsymbol{\theta}_{0C})'\boldsymbol{\Omega}^C(\boldsymbol{\theta}_{0C})^{-1}\sum_{j=1}^n \boldsymbol{U}_j^C(\boldsymbol{\theta}_{0C}) + o_p(1),
\end{aligned}$$

where

$$\begin{aligned}
\boldsymbol{D}_i^C(t;\boldsymbol{\theta}_C) &= \int_0^t \left\{\boldsymbol{Z}_i^C - \bar{\boldsymbol{z}}^C(u;\boldsymbol{\theta}_C)\right\} d\Lambda_i^C(u;\boldsymbol{\theta}_C), \\
\boldsymbol{\Omega}^C(\boldsymbol{\theta}_C) &= \mathbb{E}\bigg[\int_0^\tau \left\{\frac{\boldsymbol{s}_C^{(2)}(t;\boldsymbol{\theta}_C)}{s_C^{(0)}(t;\boldsymbol{\theta}_C)} - \bar{\boldsymbol{z}}^C(t;\boldsymbol{\theta}_C)^{\otimes 2}\right\} dM_i^C(t)\bigg].
\end{aligned}$$

Using the definition of counting processes and martingales,

$$\begin{aligned}
(4) &= \sqrt{n}\bigg\{\int_0^t \exp(\boldsymbol{\theta}_{0C}'\boldsymbol{Z}_i^C) d\widehat{\Lambda}_0^C(u;\boldsymbol{\theta}_{0C}) - \int_0^t d\Lambda_i^C(u;\boldsymbol{\theta}_{0C})\bigg\} \\
&= \sqrt{n}\bigg\{\int_0^t \exp(\boldsymbol{\theta}_{0C}'\boldsymbol{Z}_i^C)\frac{\frac{1}{n}\sum_{j=1}^n dN_j^C(u)}{\frac{1}{n}\sum_{j=1}^n R_j(u)\exp(\boldsymbol{\theta}_{0C}'\boldsymbol{Z}_j^C)} - \int_0^t d\Lambda_i^C(u;\boldsymbol{\theta}_{0C})\bigg\} \\
&= \sqrt{n}\exp(\boldsymbol{\theta}_{0C}'\boldsymbol{Z}_i^C)\bigg\{\int_0^t \frac{\frac{1}{n}\sum_{j=1}^n dN_j^C(u)}{S_C^{(0)}(u;\boldsymbol{\theta}_{0C})} - \int_0^t \frac{\frac{1}{n}\sum_{j=1}^n R_j(u)\exp(\boldsymbol{\theta}_{0C}'\boldsymbol{Z}_j^C)}{S_C^{(0)}(u;\boldsymbol{\theta}_{0C})} d\Lambda_0^C(u;\boldsymbol{\theta}_{0C})\bigg\} \\
&= \frac{1}{\sqrt{n}}\exp(\boldsymbol{\theta}_{0C}'\boldsymbol{Z}_i^C)\int_0^t S_C^{(0)}(u;\boldsymbol{\theta}_{0C})^{-1}\sum_{j=1}^n\left\{dN_j^C(u) - R_j(u)d\Lambda_j^C(u;\boldsymbol{\theta}_{0C})\right\} \\
&= \frac{1}{\sqrt{n}}\exp(\boldsymbol{\theta}_{0C}'\boldsymbol{Z}_i^C)\int_0^t \frac{\sum_{j=1}^n dM_j^C(u)}{s_C^{(0)}(u;\boldsymbol{\theta}_{0C})} + o_p(1).
\end{aligned}$$

Therefore,

$$\sqrt{n}\left\{\widehat{\Lambda}_i^C(t;\widehat{\boldsymbol{\theta}}_C) - \Lambda_i^C(t;\boldsymbol{\theta}_{0C})\right\} = \frac{1}{\sqrt{n}}\boldsymbol{D}_i^C(t;\boldsymbol{\theta}_{0C})'\boldsymbol{\Omega}^C(\boldsymbol{\theta}_{0C})^{-1}\sum_{j=1}^n \boldsymbol{U}_j^C(\boldsymbol{\theta}_{0C}) + \frac{1}{\sqrt{n}}\exp\left(\boldsymbol{\theta}_{0C}'\boldsymbol{Z}_i^C\right)\int_0^t \frac{\sum_{j=1}^n dM_j^C(u)}{s_C^{(0)}(u;\boldsymbol{\theta}_{0C})} + o_p(1),$$

and

$$\begin{aligned}
&\sqrt{n}\Big[\left\{\widehat{\Lambda}_i^C(t+S_{ik};\widehat{\boldsymbol{\theta}}_C) - \widehat{\Lambda}_i^C(S_{ik};\widehat{\boldsymbol{\theta}}_C)\right\} - \left\{\Lambda_i^C(t+S_{ik};\boldsymbol{\theta}_{0C}) - \Lambda_i^C(S_{ik};\boldsymbol{\theta}_{0C})\right\}\Big] \\
&= \frac{1}{\sqrt{n}}\widetilde{\boldsymbol{D}}_{ik}^C(t;\boldsymbol{\theta}_{0C})'\boldsymbol{\Omega}^C(\boldsymbol{\theta}_{0C})^{-1}\sum_{j=1}^n \boldsymbol{U}_j^C(\boldsymbol{\theta}_{0C}) + \frac{1}{\sqrt{n}}\exp\left(\boldsymbol{\theta}_{0C}'\boldsymbol{Z}_i^C\right)\int_{S_{ik}}^{t+S_{ik}} \frac{\sum_{j=1}^n dM_j^C(u)}{s_C^{(0)}(u;\boldsymbol{\theta}_{0C})} + o_p(1),
\end{aligned}$$

where

$$\widetilde{\boldsymbol{D}}_{ik}^C(t;\boldsymbol{\theta}_C) = \int_{S_{ik}}^{t+S_{ik}} \left\{ \boldsymbol{Z}_i^C - \bar{\boldsymbol{z}}^C(u;\boldsymbol{\theta}_C)\right\} d\Lambda_i^C(u;\boldsymbol{\theta}_C).$$

Using the Taylor expansion of $\exp(x)$ at x, the estimated IPCWs $\widehat{W}_{ik}^C(t) = \exp\left\{\widehat{\Lambda}_i^C(t+S_{ik};\widehat{\boldsymbol{\theta}}_C) - \widehat{\Lambda}_i^C(S_{ik};\widehat{\boldsymbol{\theta}}_C)\right\}$ and the true IPCWs $W_{ik}^C(t) = \exp\left\{\Lambda_i^C(t+S_{ik};\boldsymbol{\theta}_{0C}) - \Lambda_i^C(S_{ik};\boldsymbol{\theta}_{0C})\right\}$ satisfy

$$\begin{aligned}
\sqrt{n}\left\{\widehat{W}_{ik}^C(t) - W_{ik}^C(t)\right\} &= W_{ik}^C(t)\cdot\sqrt{n}\left[\left\{\widehat{\Lambda}_i^C(t+S_{ik};\widehat{\boldsymbol{\theta}}_C) - \widehat{\Lambda}_i^C(S_{ik};\widehat{\boldsymbol{\theta}}_C)\right\} - \left\{\Lambda_i^C(t+S_{ik};\boldsymbol{\theta}_{0C}) - \Lambda_i^C(S_{ik};\boldsymbol{\theta}_{0C})\right\}\right] + o_p(1) \\
&= \frac{1}{\sqrt{n}} W_{ik}^C(t) \sum_{j=1}^n \left\{\widetilde{\boldsymbol{D}}_{ik}^C(t;\boldsymbol{\theta}_{0C})'\boldsymbol{\Omega}^C(\boldsymbol{\theta}_{0C})^{-1}\boldsymbol{U}_j^C(\boldsymbol{\theta}_{0C}) + J_{ijk}^C(t;\boldsymbol{\theta}_{0C})\right\} + o_p(1),
\end{aligned}$$

where

$$J_{ijk}^C(t;\boldsymbol{\theta}_C) = \exp\left(\boldsymbol{\theta}_C'\boldsymbol{Z}_i^C\right)\int_{S_{ik}}^{t+S_{ik}} \frac{dM_j^C(u)}{s_C^{(0)}(u;\boldsymbol{\theta}_C)}.$$

Similarly, the centered process of the estimated cumulative hazards of treatment can be written as

$$\begin{aligned}
\sqrt{n}\left\{\widehat{\Lambda}_i^T(t;\widehat{\boldsymbol{\theta}}_T) - \Lambda_i^T(t;\boldsymbol{\theta}_{0T})\right\} &= \sqrt{n}\left\{\widehat{\Lambda}_i^T(t;\widehat{\boldsymbol{\theta}}_T) - \widehat{\Lambda}_i^T(t;\boldsymbol{\theta}_{0T})\right\} &\quad (5)\\
&\quad + \sqrt{n}\left\{\widehat{\Lambda}_i^T(t;\boldsymbol{\theta}_{0T}) - \Lambda_i^T(t;\boldsymbol{\theta}_{0T})\right\}. &\quad (6)
\end{aligned}$$

Using the Taylor expansion of $\widehat{\Lambda}_i^T(t;\widehat{\boldsymbol{\theta}}_T)$ at $\boldsymbol{\theta}_{0T}$,

$$\begin{aligned}
(5) &= \frac{\partial}{\partial\boldsymbol{\theta}_{0T}'}\widehat{\Lambda}_i^T(t;\boldsymbol{\theta}_{0T})\cdot\sqrt{n}\big(\widehat{\boldsymbol{\theta}}_T - \boldsymbol{\theta}_{0T}\big) + o_p(1) \\
&= \int_0^t \left\{\boldsymbol{Z}_i^T(u) - \bar{\boldsymbol{z}}^T(u;\boldsymbol{\theta}_{0T})\right\}' d\Lambda_i^T(u;\boldsymbol{\theta}_{0T})\cdot\sqrt{n}\big(\widehat{\boldsymbol{\theta}}_T - \boldsymbol{\theta}_{0T}\big) + o_p(1) \\
&= \frac{1}{\sqrt{n}}\boldsymbol{D}_i^T(t;\boldsymbol{\theta}_{0T})'\boldsymbol{\Omega}^T(\boldsymbol{\theta}_{0T})^{-1}\sum_{j=1}^n \boldsymbol{U}_j^T(\boldsymbol{\theta}_{0T}) + o_p(1),
\end{aligned}$$

where

$$\begin{aligned}
\boldsymbol{D}_i^T(t;\boldsymbol{\theta}_T) &= \int_0^t \left\{\boldsymbol{Z}_i^T(u) - \bar{\boldsymbol{z}}^T(u;\boldsymbol{\theta}_T)\right\} d\Lambda_i^T(u;\boldsymbol{\theta}_T), \\
\boldsymbol{\Omega}^T(\boldsymbol{\theta}_T) &= \mathbb{E}\left[\int_0^\tau \left\{\frac{\boldsymbol{s}_T^{(2)}(t;\boldsymbol{\theta}_T)}{s_T^{(0)}(t;\boldsymbol{\theta}_T)} - \bar{\boldsymbol{z}}^T(t;\boldsymbol{\theta}_T)^{\otimes 2}\right\} dM_i^T(t)\right].
\end{aligned}$$

Using the definition of counting processes and martingales,

$$\begin{aligned}
(6) &= \sqrt{n}\left\{\int_0^t \exp\left\{\boldsymbol{\theta}_{0T}'\boldsymbol{Z}_i^T(u)\right\} d\widehat{\Lambda}_0^T(u;\boldsymbol{\theta}_{0T}) - \int_0^t d\Lambda_i^T(u;\boldsymbol{\theta}_{0T})\right\} \\
&= \sqrt{n}\left\{\int_0^t \exp\left\{\boldsymbol{\theta}_{0T}'\boldsymbol{Z}_i^T(u)\right\} \frac{\frac{1}{n}\sum_{j=1}^n dN_j^T(u)}{\frac{1}{n}\sum_{j=1}^n R_j(u)\exp\left\{\boldsymbol{\theta}_{0T}'\boldsymbol{Z}_j^T(u)\right\}} - \int_0^t d\Lambda_i^T(u;\boldsymbol{\theta}_{0T})\right\} \\
&= \sqrt{n}\int_0^t \exp\left\{\boldsymbol{\theta}_{0T}'\boldsymbol{Z}_i^T(u)\right\}\left\{\frac{\frac{1}{n}\sum_{j=1}^n dN_j^T(u)}{S_T^{(0)}(u;\boldsymbol{\theta}_{0T})} - \frac{\frac{1}{n}\sum_{j=1}^n R_j(u)\exp\left\{\boldsymbol{\theta}_{0T}'\boldsymbol{Z}_j^T(u)\right\}}{S_T^{(0)}(u;\boldsymbol{\theta}_{0T})} d\Lambda_0^T(u;\boldsymbol{\theta}_{0T})\right\} \\
&= \frac{1}{\sqrt{n}}\int_0^t \exp\left\{\boldsymbol{\theta}_{0T}'\boldsymbol{Z}_i^T(u)\right\} S_T^{(0)}(u;\boldsymbol{\theta}_{0T})^{-1}\sum_{j=1}^n\left\{dN_j^T(u) - R_j(u)d\Lambda_j^T(u;\boldsymbol{\theta}_{0T})\right\}
\end{aligned}$$

$$= \frac{1}{\sqrt{n}} \int_0^t \exp\left\{\boldsymbol{\theta}_{0T}' \boldsymbol{Z}_i^T(u)\right\} \frac{\sum_{j=1}^n dM_j^T(u)}{s_T^{(0)}(u;\boldsymbol{\theta}_{0T})} + o_p(1).$$

Therefore,

$$\sqrt{n}\left\{\widehat{\Lambda}_i^T(t;\widehat{\boldsymbol{\theta}}_T) - \Lambda_i^T(t;\boldsymbol{\theta}_{0T})\right\} = \frac{1}{\sqrt{n}} \boldsymbol{D}_i^T(t;\boldsymbol{\theta}_{0T})' \boldsymbol{\Omega}^T(\boldsymbol{\theta}_{0T})^{-1} \sum_{j=1}^n \boldsymbol{U}_j^T(\boldsymbol{\theta}_{0T}) + \frac{1}{\sqrt{n}} \int_0^t \exp\left\{\boldsymbol{\theta}_{0T}' \boldsymbol{Z}_i^T(u)\right\} \frac{\sum_{j=1}^n dM_j^T(u)}{s_T^{(0)}(u;\boldsymbol{\theta}_{0T})} + o_p(1),$$

and

$$\begin{aligned}
&\sqrt{n}\Big[\Big\{\widehat{\Lambda}_i^T(t+S_{ik};\widehat{\boldsymbol{\theta}}_T) - \widehat{\Lambda}_i^T(S_{ik};\widehat{\boldsymbol{\theta}}_T)\Big\} - \Big\{\Lambda_i^T(t+S_{ik};\boldsymbol{\theta}_{0T}) - \Lambda_i^C(S_{ik};\boldsymbol{\theta}_{0T})\Big\}\Big] \\
&= \frac{1}{\sqrt{n}} \widetilde{\boldsymbol{D}}_{ik}^T(t;\boldsymbol{\theta}_{0T})' \boldsymbol{\Omega}^T(\boldsymbol{\theta}_{0T})^{-1} \sum_{j=1}^n \boldsymbol{U}_j^T(\boldsymbol{\theta}_{0T}) + \frac{1}{\sqrt{n}} \int_{S_{ik}}^{t+S_{ik}} \exp\left(\boldsymbol{\theta}_{0T}' \boldsymbol{Z}_i^T(u)\right) \frac{\sum_{j=1}^n dM_j^T(u)}{s_T^{(0)}(u;\boldsymbol{\theta}_{0T})} + o_p(1),
\end{aligned}$$

where

$$\widetilde{\boldsymbol{D}}_{ik}^T(t;\boldsymbol{\theta}_T) = \int_{S_{ik}}^{t+S_{ik}} \left\{\boldsymbol{Z}_i^T(u) - \bar{\boldsymbol{z}}^T(u;\boldsymbol{\theta}_T)\right\} d\Lambda_i^T(u;\boldsymbol{\theta}_T).$$

Using the Taylor expansion of $\exp(x)$ at x, the estimated IPTWs $\widehat{W}_{ik}^T(t) = \exp\left\{\widehat{\Lambda}_i^T(t+S_{ik};\widehat{\boldsymbol{\theta}}_T) - \widehat{\Lambda}_i^T(S_{ik};\widehat{\boldsymbol{\theta}}_T)\right\}$ and the true IPTWs $W_{ik}^T(t) = \exp\left\{\Lambda_i^T(t+S_{ik};\boldsymbol{\theta}_{0T}) - \Lambda_i^T(S_{ik};\boldsymbol{\theta}_{0T})\right\}$ satisfy

$$\begin{aligned}
\sqrt{n}\left\{\widehat{W}_{ik}^T(t) - W_{ik}^T(t)\right\} &= W_{ik}^T(t)\cdot\sqrt{n}\left[\left\{\widehat{\Lambda}_i^T(t+S_{ik};\widehat{\boldsymbol{\theta}}_T) - \widehat{\Lambda}_i^T(S_{ik};\widehat{\boldsymbol{\theta}}_T)\right\} - \left\{\Lambda_i^T(t+S_{ik};\boldsymbol{\theta}_{0T}) - \Lambda_i^T(S_{ik};\boldsymbol{\theta}_{0T})\right\}\right] + o_p(1) \\
&= \frac{1}{\sqrt{n}} W_{ik}^T(t) \sum_{j=1}^n \left\{\widetilde{\boldsymbol{D}}_{ik}^T(t;\boldsymbol{\theta}_{0T})' \boldsymbol{\Omega}^T(\boldsymbol{\theta}_{0T})^{-1} \boldsymbol{U}_j^T(\boldsymbol{\theta}_{0T}) + J_{ijk}^T(t;\boldsymbol{\theta}_{0T})\right\} + o_p(1),
\end{aligned}$$

where

$$J_{ijk}^T(t;\boldsymbol{\theta}_T) = \int_{S_{ik}}^{t+S_{ik}} \exp\left\{\boldsymbol{\theta}_T' \boldsymbol{Z}_i^T(u)\right\} \frac{dM_j^T(u)}{s_T^{(0)}(u;\boldsymbol{\theta}_T)}.$$

A.5 Consistency and Asymptotic Normality of $\widehat{\boldsymbol{\beta}}$

The unbiased estimating equations of the stratified landmark RMST model can be written as

$$\sum_{k=1}^K \sum_{i=1}^n R_{ik} A_{ik} \big\{\boldsymbol{Z}_{ik} - \bar{\boldsymbol{Z}}_k(\boldsymbol{\beta}, \boldsymbol{W}_k)\big\} W_{ik} \Delta_{ik}^Y \big\{Y_{ik} - \mu_{ik}(\boldsymbol{Z}_{ik};\boldsymbol{\beta})\big\} = \boldsymbol{0}, \tag{7}$$

where

$$\bar{\boldsymbol{Z}}_k(\boldsymbol{\beta}, \boldsymbol{W}_k) = \begin{cases} \dfrac{\sum_{i=1}^n R_{ik} A_{ik} W_{ik} \Delta_{ik}^Y \boldsymbol{Z}_{ik}}{\sum_{i=1}^n R_{ik} A_{ik} W_{ik} \Delta_{ik}^Y} & \text{if } \mu_{ik}(\boldsymbol{Z}_{ik};\boldsymbol{\beta}) = \mu_{0k} + \boldsymbol{\beta}' \boldsymbol{Z}_{ik} \\ \dfrac{\sum_{i=1}^n R_{ik} A_{ik} W_{ik} \Delta_{ik}^Y \exp(\boldsymbol{\beta}' \boldsymbol{Z}_{ik}) \boldsymbol{Z}_{ik}}{\sum_{i=1}^n R_{ik} A_{ik} W_{ik} \Delta_{ik}^Y \exp(\boldsymbol{\beta}' \boldsymbol{Z}_{ik})} & \text{if } \mu_{ik}(\boldsymbol{Z}_{ik};\boldsymbol{\beta}) = \mu_{0k} \exp(\boldsymbol{\beta}' \boldsymbol{Z}_{ik}) \end{cases}.$$

Define

$$\boldsymbol{\phi}(\boldsymbol{Y}_i, \boldsymbol{Z}_i; \boldsymbol{\beta}, \boldsymbol{\mu}_0) = \sum_{k=1}^K R_{ik} A_{ik} \left\{\boldsymbol{Z}_{ik} - \bar{\boldsymbol{Z}}_k(\boldsymbol{\beta}, \widehat{\boldsymbol{W}}_k)\right\} \widehat{W}_{ik} \Delta_{ik}^Y \left\{Y_{ik} - \mu_{ik}(\boldsymbol{Z}_{ik};\boldsymbol{\beta})\right\}, \text{ and}$$

$$\boldsymbol{\Phi}_n(\boldsymbol{\beta}) = \frac{1}{n} \sum_{i=1}^n \boldsymbol{\phi}(\boldsymbol{Y}_i, \boldsymbol{Z}_i; \boldsymbol{\beta}, \boldsymbol{\mu}_0) = \frac{1}{n} \sum_{i=1}^n \sum_{k=1}^K R_{ik} A_{ik} \left\{\boldsymbol{Z}_{ik} - \bar{\boldsymbol{Z}}_k(\boldsymbol{\beta}, \widehat{\boldsymbol{W}}_k)\right\} \widehat{W}_{ik} \Delta_{ik}^Y \left\{Y_{ik} - \mu_{ik}(\boldsymbol{Z}_{ik};\boldsymbol{\beta})\right\},$$

where $\boldsymbol{Y}_i = (Y_{ik})_{k=1,\ldots,K}$, $\boldsymbol{Z}_i = (\boldsymbol{Z}_{ik})_{k=1,\ldots,K}$, $\boldsymbol{\mu}_0 = (\mu_{0k})_{k=1,\ldots,K}$, and $\widehat{\boldsymbol{W}} = (\widehat{\boldsymbol{W}}_k)_{k=1,\ldots,K}$.

$\sqrt{n}\boldsymbol{\Phi}_n(\boldsymbol{\beta})$ can be written as

$$
\begin{aligned}
\sqrt{n}\boldsymbol{\Phi}_n(\boldsymbol{\beta}) &= \frac{1}{\sqrt{n}}\sum_{i=1}^{n}\sum_{k=1}^{K} R_{ik}A_{ik}\left\{\boldsymbol{Z}_{ik} - \bar{\boldsymbol{Z}}_k(\boldsymbol{\beta}, \widehat{\boldsymbol{W}}_k)\right\}\widehat{W}^C_{ik}(Y_{ik})\widehat{W}^T_{ik}(Y_{ik})\Delta^Y_{ik}\left\{Y_{ik} - \mu_{ik}(\boldsymbol{Z}_{ik};\boldsymbol{\beta})\right\} \\
&= \frac{1}{\sqrt{n}}\sum_{i=1}^{n}\sum_{k=1}^{K} R_{ik}A_{ik}\left\{\boldsymbol{Z}_{ik} - \bar{\boldsymbol{Z}}_k(\boldsymbol{\beta}, \boldsymbol{W}_k) + \bar{\boldsymbol{Z}}_k(\boldsymbol{\beta}, \boldsymbol{W}_k) - \bar{\boldsymbol{Z}}_k(\boldsymbol{\beta}, \widehat{\boldsymbol{W}}_k)\right\} \\
&\qquad \cdot\left\{\widehat{W}^C_{ik}(Y_{ik}) - W^C_{ik}(Y_{ik}) + W^C_{ik}(Y_{ik})\right\}\left\{(\widehat{W}^T_{ik}(Y_{ik}) - W^T_{ik}(Y_{ik}) + W^T_{ik}(Y_{ik})\right\}\Delta^Y_{ik}\left\{Y_{ik} - \mu_{ik}(\boldsymbol{Z}_{ik};\boldsymbol{\beta})\right\} \\
&= \frac{1}{\sqrt{n}}\sum_{i=1}^{n}\sum_{k=1}^{K} R_{ik}A_{ik}\left\{\boldsymbol{Z}_{ik} - \bar{\boldsymbol{Z}}_k(\boldsymbol{\beta}, \boldsymbol{W}_k)\right\}W^C_{ik}(Y_{ik})W^T_{ik}(Y_{ik})\Delta^Y_{ik}\left\{Y_{ik} - \mu_{ik}(\boldsymbol{Z}_{ik};\boldsymbol{\beta})\right\} \qquad (8) \\
&+ \frac{1}{\sqrt{n}}\sum_{i=1}^{n}\sum_{k=1}^{K} R_{ik}A_{ik}\left\{\boldsymbol{Z}_{ik} - \bar{\boldsymbol{Z}}_k(\boldsymbol{\beta}, \boldsymbol{W}_k)\right\}\left\{\widehat{W}^C_{ik}(Y_{ik}) - W^C_{ik}(Y_{ik})\right\}\left\{\widehat{W}^T_{ik}(Y_{ik}) - W^T_{ik}(Y_{ik})\right\} \\
&\qquad \cdot\Delta^Y_{ik}\left\{Y_{ik} - \mu_{ik}(\boldsymbol{Z}_{ik};\boldsymbol{\beta})\right\} \qquad (9) \\
&+ \frac{1}{\sqrt{n}}\sum_{i=1}^{n}\sum_{k=1}^{K} R_{ik}A_{ik}\left\{\boldsymbol{Z}_{ik} - \bar{\boldsymbol{Z}}_k(\boldsymbol{\beta}, \boldsymbol{W}_k)\right\}W^C_{ik}(Y_{ik})\left\{\widehat{W}^T_{ik}(Y_{ik}) - W^T_{ik}(Y_{ik})\right\}\Delta^Y_{ik}\left\{Y_{ik} - \mu_{ik}(\boldsymbol{Z}_{ik};\boldsymbol{\beta})\right\} \\
&\qquad (10) \\
&+ \frac{1}{\sqrt{n}}\sum_{i=1}^{n}\sum_{k=1}^{K} R_{ik}A_{ik}\left\{\boldsymbol{Z}_{ik} - \bar{\boldsymbol{Z}}_k(\boldsymbol{\beta}, \boldsymbol{W}_k)\right\}W^T_{ik}(Y_{ik})\left\{\widehat{W}^C_{ik}(Y_{ik}) - W^C_{ik}(Y_{ik})\right\}\Delta^Y_{ik}\left\{Y_{ik} - \mu_{ik}(\boldsymbol{Z}_{ik};\boldsymbol{\beta})\right\} \\
&\qquad (11) \\
&+ \frac{1}{\sqrt{n}}\sum_{i=1}^{n}\sum_{k=1}^{K} R_{ik}A_{ik}\left\{\bar{\boldsymbol{Z}}_k(\boldsymbol{\beta}, \boldsymbol{W}_k) - \bar{\boldsymbol{Z}}_k(\boldsymbol{\beta}, \widehat{\boldsymbol{W}}_k)\right\}\widehat{W}^C_{ik}(Y_{ik})\widehat{W}^T_{ik}(Y_{ik})\Delta^Y_{ik}\left\{Y_{ik} - \mu_{ik}(\boldsymbol{Z}_{ik};\boldsymbol{\beta})\right\}. \qquad (12)
\end{aligned}
$$

Based on regularity condition (iv), $\widehat{W}^C_{ik}(t) \xrightarrow{p} W^C_{ik}(t)$ and $\widehat{W}^T_{ik}(t) \xrightarrow{p} W^T_{ik}(t)$. By the continuous mapping theorem, (12) converges to 0 in probability.

Let $\boldsymbol{\epsilon}_{ik}(\boldsymbol{\beta}) = \left\{\boldsymbol{Z}_{ik} - \bar{\boldsymbol{Z}}_k(\boldsymbol{\beta}, \boldsymbol{W}_k)\right\}W^C_{ik}(Y_{ik})W^T_{ik}(Y_{ik})\Delta^Y_{ik}\left\{Y_{ik} - \mu_{ik}(\boldsymbol{Z}_{ik};\boldsymbol{\beta})\right\}$. Then,

$$
\begin{aligned}
(8) &= \frac{1}{\sqrt{n}}\sum_{i=1}^{n}\sum_{k=1}^{K} R_{ik}A_{ik}\boldsymbol{\epsilon}_{ik}(\boldsymbol{\beta}), \\
(9) &= \frac{1}{\sqrt{n}}\sum_{i=1}^{n}\sum_{k=1}^{K} R_{ik}A_{ik}\boldsymbol{\epsilon}_{ik}(\boldsymbol{\beta})\frac{\widehat{W}^C_{ik}(Y_{ik}) - W^C_{ik}(Y_{ik})}{W^C_{ik}(Y_{ik})}\frac{\widehat{W}^T_{ik}(Y_{ik}) - W^T_{ik}(Y_{ik})}{W^T_{ik}(Y_{ik})} \\
&= \frac{1}{n^{5/2}}\sum_{i=1}^{n}\sum_{k=1}^{K} R_{ik}A_{ik}\boldsymbol{\epsilon}_{ik}(\boldsymbol{\beta})\sum_{j=1}^{n}\left\{\widetilde{\boldsymbol{D}}^C_{ik}(Y_{ik};\boldsymbol{\theta}_{0C})'\boldsymbol{\Omega}^C(\boldsymbol{\theta}_{0C})^{-1}\boldsymbol{U}^C_j(\boldsymbol{\theta}_{0C}) + J^C_{ijk}(Y_{ik};\boldsymbol{\theta}_{0C})\right\} \\
&\qquad \cdot\sum_{j'=1}^{n}\left\{\widetilde{\boldsymbol{D}}^T_{ik}(Y_{ik};\boldsymbol{\theta}_{0T})'\boldsymbol{\Omega}^T(\boldsymbol{\theta}_{0T})^{-1}\boldsymbol{U}^T_{j'}(\boldsymbol{\theta}_{0T}) + J^T_{ij'k}(Y_{ik};\boldsymbol{\theta}_{0T})\right\} + o_p(1), \\
(10) &= \frac{1}{\sqrt{n}}\sum_{i=1}^{n}\sum_{k=1}^{K} R_{ik}A_{ik}\boldsymbol{\epsilon}_{ik}(\boldsymbol{\beta})\frac{\widehat{W}^T_{ik}(Y_{ik}) - W^T_{ik}(Y_{ik})}{W^T_{ik}(Y_{ik})} \\
&= \frac{1}{n^{3/2}}\sum_{i=1}^{n}\sum_{k=1}^{K} R_{ik}A_{ik}\boldsymbol{\epsilon}_{ik}(\boldsymbol{\beta})\sum_{j=1}^{n}\left\{\widetilde{\boldsymbol{D}}^T_{ik}(Y_{ik};\boldsymbol{\theta}_{0T})'\boldsymbol{\Omega}^T(\boldsymbol{\theta}_{0T})^{-1}\boldsymbol{U}^T_j(\boldsymbol{\theta}_{0T}) + J^T_{ijk}(Y_{ik};\boldsymbol{\theta}_{0T})\right\} + o_p(1),
\end{aligned}
$$

$$(11) = \frac{1}{\sqrt{n}} \sum_{i=1}^{n} \sum_{k=1}^{K} R_{ik} A_{ik} \boldsymbol{\epsilon}_{ik}(\boldsymbol{\beta}) \frac{\widehat{W}_{ik}^{C}(Y_{ik}) - W_{ik}^{C}(Y_{ik})}{W_{ik}^{C}(Y_{ik})}$$

$$= \frac{1}{n^{3/2}} \sum_{i=1}^{n} \sum_{k=1}^{K} R_{ik} A_{ik} \boldsymbol{\epsilon}_{ik}(\boldsymbol{\beta}) \sum_{j=1}^{n} \Big\{ \widetilde{\boldsymbol{D}}_{ik}^{C}(Y_{ik}; \boldsymbol{\theta}_{0C})' \boldsymbol{\Omega}^{C}(\boldsymbol{\theta}_{0C})^{-1} \boldsymbol{U}_{j}^{C}(\boldsymbol{\theta}_{0C}) + J_{ijk}^{C}(Y_{ik}; \boldsymbol{\theta}_{0C}) \Big\} + o_p(1).$$

(9) only contains second-order terms which converge fast enough towards zero and hence is of negligible importance.

Let

$$\boldsymbol{k}^{C}(\boldsymbol{\beta}, \boldsymbol{\theta}_{C}) = \mathbb{E}\Big\{ \sum_{k=1}^{K} R_{ik} A_{ik} \boldsymbol{\epsilon}_{ik}(\boldsymbol{\beta}) \widetilde{\boldsymbol{D}}_{ik}^{C}(Y_{ik}; \boldsymbol{\theta}_{C})' \Big\},$$

$$\boldsymbol{h}^{C}(t; \boldsymbol{\beta}, \boldsymbol{\theta}_{C}) = \mathbb{E}\Big\{ \sum_{k=1}^{K} R_{ik} A_{ik} \boldsymbol{\epsilon}_{ik}(\boldsymbol{\beta}) I(S_{ik} \leqslant t \leqslant S_{ik} + L) R_i(t) \exp\big(\boldsymbol{\theta}_{C}' \boldsymbol{Z}_{i}^{C} \big) \Big\},$$

$$\boldsymbol{k}^{T}(\boldsymbol{\beta}, \boldsymbol{\theta}_{T}) = \mathbb{E}\Big\{ \sum_{k=1}^{K} R_{ik} A_{ik} \boldsymbol{\epsilon}_{ik}(\boldsymbol{\beta}) \widetilde{\boldsymbol{D}}_{ik}^{T}(Y_{ik}; \boldsymbol{\theta}_{T})' \Big\},$$

$$\boldsymbol{h}^{T}(t; \boldsymbol{\beta}, \boldsymbol{\theta}_{T}) = \mathbb{E}\Big[\sum_{k=1}^{K} R_{ik} A_{ik} \boldsymbol{\epsilon}_{ik}(\boldsymbol{\beta}) I(S_{ik} \leqslant t \leqslant S_{ik} + L) R_i(t) \exp\big\{ \boldsymbol{\theta}_{T}' \boldsymbol{Z}_{i}^{T}(t) \big\} \Big].$$

Then

$$(10) = \frac{1}{n^{3/2}} \sum_{i=1}^{n} \sum_{k=1}^{K} R_{ik} A_{ik} \boldsymbol{\epsilon}_{ik}(\boldsymbol{\beta}) \sum_{j=1}^{n} \Big\{ \widetilde{\boldsymbol{D}}_{ik}^{T}(Y_{ik}; \boldsymbol{\theta}_{0T})' \boldsymbol{\Omega}^{T}(\boldsymbol{\theta}_{0T})^{-1} \boldsymbol{U}_{j}^{T}(\boldsymbol{\theta}_{0T}) + J_{ijk}^{T}(Y_{ik}; \boldsymbol{\theta}_{0T}) \Big\} + o_p(1)$$

$$= \frac{1}{\sqrt{n}} \boldsymbol{k}^{T}(\boldsymbol{\beta}, \boldsymbol{\theta}_{0T}) \boldsymbol{\Omega}^{T}(\boldsymbol{\theta}_{0T})^{-1} \sum_{i=1}^{n} \boldsymbol{U}_{i}^{T}(\boldsymbol{\theta}_{0T}) + \frac{1}{\sqrt{n}} \int_0^{\tau} \frac{\boldsymbol{h}^{T}(t; \boldsymbol{\beta}, \boldsymbol{\theta}_{0T}) \sum_{i=1}^{n} dM_i^{T}(t)}{s_T^{(0)}(t; \boldsymbol{\theta}_{0T})} + o_p(1),$$

$$(11) = \frac{1}{n^{3/2}} \sum_{i=1}^{n} \sum_{k=1}^{K} R_{ik} A_{ik} \boldsymbol{\epsilon}_{ik}(\boldsymbol{\beta}) \sum_{j=1}^{n} \Big\{ \widetilde{\boldsymbol{D}}_{ik}^{C}(Y_{ik}; \boldsymbol{\theta}_{0C})' \boldsymbol{\Omega}^{C}(\boldsymbol{\theta}_{0C})^{-1} \boldsymbol{U}_{j}^{C}(\boldsymbol{\theta}_{0C}) + J_{ijk}^{C}(Y_{ik}; \boldsymbol{\theta}_{0C}) \Big\} + o_p(1)$$

$$= \frac{1}{\sqrt{n}} \boldsymbol{k}^{C}(\boldsymbol{\beta}, \boldsymbol{\theta}_{0C}) \boldsymbol{\Omega}^{C}(\boldsymbol{\theta}_{0C})^{-1} \sum_{i=1}^{n} \boldsymbol{U}_{i}^{C}(\boldsymbol{\theta}_{0C}) + \frac{1}{\sqrt{n}} \int_0^{\tau} \frac{\boldsymbol{h}^{C}(t; \boldsymbol{\beta}, \boldsymbol{\theta}_{0C}) \sum_{i=1}^{n} dM_i^{C}(t)}{s_C^{(0)}(t; \boldsymbol{\theta}_{0C})} + o_p(1).$$

Therefore, $\sqrt{n}\boldsymbol{\Phi}_n(\boldsymbol{\beta})$ can be written as $\sqrt{n}\boldsymbol{\Phi}_n(\boldsymbol{\beta}) = \frac{1}{\sqrt{n}} \sum_{i=1}^{n} \boldsymbol{B}_i(\boldsymbol{\beta}) + o_p(1)$, where

$$\boldsymbol{B}_i(\boldsymbol{\beta}) = \sum_{k=1}^{K} R_{ik} A_{ik} \boldsymbol{\epsilon}_{ik}(\boldsymbol{\beta}) + \boldsymbol{k}^{T}(\boldsymbol{\beta}, \boldsymbol{\theta}_{0T}) \boldsymbol{\Omega}^{T}(\boldsymbol{\theta}_{0T})^{-1} \boldsymbol{U}_{i}^{T}(\boldsymbol{\theta}_{0T}) + \int_0^{\tau} \frac{\boldsymbol{h}^{T}(t; \boldsymbol{\beta}, \boldsymbol{\theta}_{0T}) dM_i^{T}(t)}{s_T^{(0)}(t; \boldsymbol{\theta}_{0T})}$$
$$+ \boldsymbol{k}^{C}(\boldsymbol{\beta}, \boldsymbol{\theta}_{0C}) \boldsymbol{\Omega}^{C}(\boldsymbol{\theta}_{0C})^{-1} \boldsymbol{U}_{i}^{C}(\boldsymbol{\theta}_{0C}) + \int_0^{\tau} \frac{\boldsymbol{h}^{C}(t; \boldsymbol{\beta}, \boldsymbol{\theta}_{0C}) dM_i^{C}(t)}{s_C^{(0)}(t; \boldsymbol{\theta}_{0C})}.$$

Define $\boldsymbol{B}(\boldsymbol{\beta}_0) = \mathbb{E}\big\{ \boldsymbol{B}_i(\boldsymbol{\beta}_0)^{\otimes 2} \big\}$. By the Central Limit Theorem, $\sqrt{n}\boldsymbol{\Phi}_n(\boldsymbol{\beta}_0) \xrightarrow{D} \mathcal{N}(\boldsymbol{0}, \boldsymbol{B}(\boldsymbol{\beta}_0))$.

To derive the asymptotic distribution of $\widehat{\boldsymbol{\beta}}$, a Taylor expansion of $\boldsymbol{\Phi}_n(\widehat{\boldsymbol{\beta}})$ about $\boldsymbol{\beta}_0$ yields

$$\sqrt{n}\big(\widehat{\boldsymbol{\beta}} - \boldsymbol{\beta}_0\big) = \Big\{ -\frac{\partial \boldsymbol{\Phi}_n(\boldsymbol{\beta}_0)}{\partial \boldsymbol{\beta}_0} \Big\}^{-1} \sqrt{n}\boldsymbol{\Phi}_n(\boldsymbol{\beta}_0) + o_p(1).$$

Define $\boldsymbol{A}(\boldsymbol{\beta}) = \mathbb{E}\Big\{ -\partial\boldsymbol{\Phi}_n(\boldsymbol{\beta})/\partial\boldsymbol{\beta}\Big\}$, where

$$-\frac{\partial\boldsymbol{\Phi}_n(\boldsymbol{\beta})}{\partial\boldsymbol{\beta}} = \begin{cases} \frac{1}{n}\sum_{i=1}^{n}\sum_{k=1}^{K} R_{ik}A_{ik}\widehat{W}_{ik}\Delta_{ik}^{Y}(\boldsymbol{Z}_{ik} - \bar{\boldsymbol{z}}_k^{A})^{\otimes 2} & \text{if } \mu_{ik}(\boldsymbol{Z}_{ik};\boldsymbol{\beta}) = \mu_{0k} + \boldsymbol{\beta}'\boldsymbol{Z}_{ik} \\ \frac{1}{n}\sum_{i=1}^{n}\sum_{k=1}^{K} R_{ik}A_{ik}\widehat{W}_{ik}\Delta_{ik}^{Y}Y_{ik}\left\{\frac{s_k^{(2)}(\boldsymbol{\beta})}{s_k^{(0)}(\boldsymbol{\beta})} - \bar{\boldsymbol{z}}_k^{M}(\boldsymbol{\beta})^{\otimes 2}\right\} & \text{if } \mu_{ik}(\boldsymbol{Z}_{ik};\boldsymbol{\beta}) = \mu_{0k}\exp(\boldsymbol{\beta}'\boldsymbol{Z}_{ik}) \end{cases}.$$

Applying the delta method, $\sqrt{n}\big(\widehat{\boldsymbol{\beta}} - \boldsymbol{\beta}_0\big) \xrightarrow{D} \mathcal{N}\Big(\mathbf{0}, \boldsymbol{A}(\boldsymbol{\beta}_0)^{-1}\boldsymbol{B}(\boldsymbol{\beta}_0)\left\{\boldsymbol{A}(\boldsymbol{\beta}_0)^{-1}\right\}'\Big)$.

Appendix B Estimation Procedure for Extended Models

B.1 CS_k-Dependent Coefficients

The model with CS_k-dependent coefficients is

$$\mu_{ik}(\boldsymbol{Z}_{ik};\boldsymbol{\beta}_k) := \mu_{0k}\exp\left(\boldsymbol{\beta}_k'\boldsymbol{Z}_{ik}\right), \tag{13}$$

where $\boldsymbol{\beta}_k$ can be a vector of p arbitrary parameters or a vector of p functions of k.

If $\boldsymbol{\beta}_k$ is a vector of p arbitrary parameters, $\boldsymbol{\beta}_k$ could be estimated using the proposed methods by modifying the specification of $\boldsymbol{Z}_{ik}$ to incorporate the interaction between the cross-sections and the pre-specified covariate functionals.

Suppose $\boldsymbol{\beta}_k$ is a vector of p functions of k, which can be viewed as p linear combinations of a finite number of pre-specified functions of k. Then $\boldsymbol{\beta}_k$ can be mathematically written as $\boldsymbol{\beta}_k = \boldsymbol{\alpha}_1 f_1(k) + \cdots + \boldsymbol{\alpha}_q f_q(k)$, where $q < \infty$ is the number of pre-specified functions, and $\boldsymbol{\alpha}_1 = (\alpha_{11},\ldots,\alpha_{p1})',\ldots,\boldsymbol{\alpha}_q = (\alpha_{1q},\ldots,\alpha_{pq})'$. Let $\boldsymbol{f}(k) = (f_1(k),\ldots,f_m(k))'$. The model expression in (13) can therefore be rewritten as $\mu_{0k}\exp\left\{\widetilde{\boldsymbol{\beta}}'\widetilde{\boldsymbol{Z}}_{ik}\right\}$, where $\widetilde{\boldsymbol{\beta}} = (\boldsymbol{\alpha}_1,\ldots,\boldsymbol{\alpha}_q)'$ and $\widetilde{\boldsymbol{Z}}_{ik} = \boldsymbol{Z}_{ik}\otimes\boldsymbol{f}(k)$, with $\otimes$ denoting the Kronecker product. This new parametrization is similar to the stratified multiplicative model, and the new model parameters $\widetilde{\boldsymbol{\beta}}$ can be estimated using the proposed methods.

One should be noted that model (13) may be high-dimensional. For example, as the number of cross-sections K increases (when $\boldsymbol{\beta}_k$ is a vector of arbitrary parameters) or as the number of pre-specified functions increases (when $\boldsymbol{\beta}_k$ is specified as a vector of functions of k), the model can be too dense and computationally expensive to estimate. That is, when we assume that covariate effects are dependent on cross-sections, we may lose computational efficiency as the effect of cross-sections could not be completely factored out of the model.

B.2 S_{ik}-Dependent Coefficients

The model with S_{ik}-dependent coefficients is

$$\mu_{ik}\{\boldsymbol{Z}_{ik}, S_{ik};\boldsymbol{\beta}(S_{ik})\} := \mu_{0k}\exp\left\{\boldsymbol{\beta}(S_{ik})'\boldsymbol{Z}_{ik}\right\}, \tag{14}$$

where $\boldsymbol{\beta}(S_{ik})$ is a vector of p functions of S_{ik}.

This model again becomes a sub-type of the stratified multiplicative model by merging the part of $\boldsymbol{\beta}(S_{ik})$ that depends on S_{ik} into $\boldsymbol{Z}_{ik}$ as time-dependent covariates. With some re-parametrization the model parameters can be estimated using the proposed method.

Appendix C Additional Simulation Results

C.1 Increased Number of Cross-Sections

We increase the number of cross-sections to $K = 10$ (so that the time interval between consecutive cross-sections is reduced to $m = 5$) and evaluate the performance of our proposed methods. As K increases, the values of $\lambda_0^C(t)$ and $\lambda_0^T(t)$ need to be changed in order to maintain the specified levels of censoring considered in the main manuscript. For low censoring (15% treatment censoring and 10% administrative censoring), we set $\lambda_0^C(t) = 0.0007$ and $\lambda_0^T(t) = 0.0020$; for high censoring (30% treatment censoring and 20% administrative censoring), we set $\lambda_0^C(t) = 0.0016$ and $\lambda_0^T(t) = 0.0036$.

We show the results for the general hazards survival model which better represents the real world data. Table C.1 shows the simulation results for the scenario of low censoring, whereas Table C.2 shows the simulation results for the scenario of high censoring. In both scenarios, both the additive model and the multiplicative model yield reasonably negligible bias, asymptotic standard errors which closely approximate empirical standard deviation, and good coverage probabilities which differ only slightly from the nominal 95%. Therefore, we conclude that the proposed working models perform well for different numbers of cross-section, and a larger set of landmarks leads to more accurate and efficient parameter estimates.

Table C.1: Bias, empirical standard deviation (ESD), average asymptotic standard error (ASE), and coverage probability (CP) of the proposed estimators for $L = 50$ and $K = 10$ under general hazards model with *low censoring* (15% treatment censoring and 10% independent censoring), based on 1000 replications of $n = 2500$ samples.

**Model**	**Parameter**	**Bias**	**ESD**	**ASE**	**CP (%)**
Additive	$\beta_a = 1.30$	0.020	0.397	0.401	95.3
	$\beta_b = 0.52$	-0.001	0.022	0.023	94.7
Multiplicative	$\beta_a = 0.03$	0.000	0.009	0.009	94.8
	$\beta_b = 0.01$	0.000	0.001	0.001	95.7

Table C.2: Bias, empirical standard deviation (ESD), average asymptotic standard error (ASE), and coverage probability (CP) of the proposed estimators for $L = 50$ and $K = 10$ under general hazards model with *high censoring* (30% treatment censoring and 20% independent censoring), based on 1000 replications of $n = 2500$ samples.

**Model**	**Parameter**	**Bias**	**ESD**	**ASE**	**CP (%)**
Additive	$\beta_a = 1.30$	-0.0020	0.411	0.421	95.6
	$\beta_b = 0.52$	-0.0006	0.024	0.024	95.6
Multiplicative	$\beta_a = 0.03$	-0.0004	0.0097	0.0096	95.0
	$\beta_b = 0.01$	-0.0001	0.0006	0.0006	95.2

C.2 Relative Bias

The absolute bias from the additive model is not directly comparable to that from the multiplicative model because the underlying true parameter values are on different scales. Therefore, we report relative bias in this section to demonstrate that the bias from both working models is adequately small.

Table C.3 displays the absolute and relative biases for the additive and multiplicative models used in the simulation study. The relative bias for both models are negligible ($< 5\%$) and comparable.

Table C.3: Absolute (abs) bias and relative (rel) bias of the proposed estimators for $L = 50$ and $K = 5$, based on 1000 replications of $n = 2500$ samples.

		**Additive Model**			**Multiplicative Model**		
**Censoring**	**Hazards**	**Parameter**	**Abs bias**	**Rel bias (%)**	**Parameter**	**Abs bias**	**Rel bias (%)**
Low	General	$\beta_a = 2.75$	0.030	1.10	$\beta_a = 0.08$	-0.001	1.35
		$\beta_b = 1.21$	0.001	0.07	$\beta_b = 0.03$	0.000	0.03
	PH	$\beta_a = 3.00$	-0.022	-0.72	$\beta_a = 0.08$	0.001	1.46
		$\beta_b = 1.75$	0.004	0.24	$\beta_b = 0.05$	0.000	0.72
High	General	$\beta_a = 2.75$	-0.015	-0.53	$\beta_a = 0.08$	-0.001	-1.16
		$\beta_b = 1.21$	-0.002	-0.21	$\beta_b = 0.03$	0.000	-0.14
	PH	$\beta_a = 3.00$	0.017	0.58	$\beta_a = 0.08$	0.001	1.95
		$\beta_b = 1.75$	0.021	1.18	$\beta_b = 0.05$	0.001	2.76

Appendix D Additional Data Analysis Results

D.1 Treatment Model

Table D.1: Estimation of the Cox proportional hazards treatment model: parameter estimate ($\widehat{\boldsymbol{\theta}}_T$), robust standard error (SE), and p-value.

	$\widehat{\boldsymbol{\theta}}_T$	robust SE	p-value
Time-fixed/baseline covariates			
Age/5 (years)	0.00	0.005	0.99
Sex (Female)	-0.08	0.021	0.0003
Diabetes (Yes)	0.03	0.016	0.06
Liver diagnosis (reference: non-cholestatic cirrhosis)			
Hepatitis C	-0.02	0.019	0.31
Cholestatic cirrhosis	0.22	0.025	< 0.0001
Acute hepatic nephropathy	-0.36	0.229	0.12
Metastatic disease	-0.07	0.059	0.20
Malignant neoplasm	0.02	0.023	0.42
Race (reference: White)			
Black	-0.02	0.025	0.34
Hispanic	-0.09	0.023	0.0002
Asian	-0.03	0.036	0.40
Other	0.01	0.049	0.88
Malignancy (Yes)	0.08	0.024	0.002
Height/10 (cm)	0.06	0.010	< 0.0001
Weight/5 (kg)	0.002	0.002	0.32
Functional status	0.01	0.005	0.03
Working for income	0.08	0.017	< 0.0001
Time-dependent covariates			
Dialysis (Yes)	-0.32	0.037	< 0.0001
Allocation MELD score	0.17	0.002	< 0.0001
Ascites	0.02	0.017	0.23
Encephalopathy	0.10	0.015	< 0.0001
Albumin	0.01	0.012	0.53
Sodium	-0.03	0.003	< 0.0001

We summarize the estimation and inference results of the treatment model in Table D.1. The significant baseline covariates are sex, liver disease diagnosis (cholestatic cirrhosis as compared to non-cholestatic cirrhosis), race (Hispanic as compared to White), malignancy, height, functional status and whether the patient was working for income. The significant time-dependent covariates are use of dialysis, allocation MELD score, encephalopathy, and serum sodium.

D.2 Covariate Effects Varied with L

We illustrate the problem using quarterly cross-sections from calendar years 2010 to 2019 ($K = 40$) as an example. As shown in Table D.2, the effect of bilirubin, creatinine, and INR when the restriction time is chosen to be $L = 30$

or 90 *days* is negative as expected in the MELD score formula, which is consistent with the fact that patients with a higher MELD score are more likely to die without a liver transplant. However, their effect direction changes when the restriction time is chosen to be $L = 365$ *days*. A possible explanation to this observation is that the covariate values at a particular time may gradually lose predictive power for survival within a longer time period.

Table D.2: Analysis of liver transplant data: effect estimate ($\widehat{\boldsymbol{\beta}}$ in *days*), standard error (SE), and p-value of time-dependent covariates for $L = 30, 90, 180$, and 365 *days*.

	$L = 30$		$L = 90$		$L = 180$		$L = 365$	
	Estimate	p-value	Estimate	p-value	Estimate	p-value	Estimate	p-value
Time-varying covariates								
Dialysis (Yes)	-0.04	0.14	-0.20	0.42	-0.07	0.90	0.05	0.96
Ascites	0.01	0.01	0.03	0.29	0.06	0.47	0.26	0.30
Encephalopathy	-0.005	0.37	-0.03	0.27	-0.06	0.47	-0.07	0.80
Albumin	0.01	0.05	0.20	< 0.0001	0.82	< 0.0001	2.37	< 0.0001
Bilirubin	-0.04	< 0.0001	-0.08	< 0.0001	0.03	0.56	0.47	0.002
Creatinine	-0.14	< 0.0001	-0.37	0.003	-0.36	0.29	0.82	0.30
INR	-0.15	< 0.0001	-0.44	0.003	-0.22	0.51	1.51	0.02
Serum sodium	0.005	< 0.0001	0.02	< 0.0001	0.05	< 0.0001	0.09	0.001
Follow-up time	0.000001	0.71	-0.00008	< 0.0001	-0.0004	< 0.0001	-0.001	< 0.0001

D.3 Finer Grid of Landmark Dates

In the main manuscript, we considered cross-section landmark dates monthly ($K = 120$). We can also set landmark dates with longer intervals, for example, annually ($K = 10$), semiannually ($K = 20$), or quarterly ($K = 40$), or set landmark dates with shorter intervals, for example, weekly ($K = 520$). In Table D.3, we tabulate analysis results for these K values using the additive model. The estimates are fairly consistent across different K's. Covariate effects generally become more significant as more cross-section dates are considered, which is as expected because more data points are included in the stacked dataset.

References

Table D.3: Analysis of liver transplant data: effect estimate ($\widehat{\boldsymbol{\beta}}$ in *days*) and p-value for $L = 90$ days.

	Annually		Semiannually		Quarterly		Monthly		Weekly	
	Estimate	p-value	Estimate	p-value	Estimate	p-value	Estimate	p-value	Estimate	p-value
Baseline covariates										
Age/5 (years)	-0.07	< 0.0001	-0.07	< 0.0001	-0.07	< 0.0001	-0.07	< 0.0001	-0.08	< 0.0001
Sex (Female)	0.03	0.56	0.04	0.39	0.07	0.03	0.09	0.002	0.09	0.002
Diabetes (Yes)	-0.2	0.001	-0.04	0.34	-0.02	0.58	-0.03	0.29	-0.02	0.39
Liver diagnosis (reference: non-cholestatic cirrhosis)										
Hepatitis C	-0.002	0.96	-0.03	0.46	-0.02	0.65	-0.02	0.33	-0.03	0.20
Cholestatic cirrhosis	0.2	0.02	0.03	0.54	0.01	0.77	0.02	0.54	-0.005	0.88
Acute hepatic nephropathy	-0.004	0.98	0.1	0.45	0.06	0.65	0.09	0.35	0.11	0.28
Metastatic disease	0.1	0.26	0.04	0.80	-0.008	0.95	-0.08	0.51	-0.08	0.50
Malignant neoplasm	-0.5	< 0.0001	-0.4	< 0.0001	-0.4	< 0.0001	-0.4	< 0.0001	-0.4	< 0.0001
Race (reference: White)										
Black	-0.04	0.59	0.04	0.52	0.07	0.13	0.04	0.22	0.05	0.15
Hispanic	0.04	0.49	0.04	0.40	0.02	0.59	0.05	0.06	0.05	0.04
Asian	0.1	0.14	0.1	0.10	0.1	0.01	0.2	< 0.0001	0.2	< 0.0001
Other	0.1	0.43	0.1	0.23	0.08	0.29	0.009	0.88	0.02	0.78
Malignancy (Yes)	0.08	0.21	0.03	0.61	-0.01	0.73	-0.07	0.05	-0.06	0.09
Height/10 (cm)	-0.04	0.24	-0.07	0.18	-0.05	0.11	-0.02	0.27	-0.01	0.41
Weight/5 (kg)	0.008	0.23	0.01	0.03	0.01	0.002	0.01	< 0.0001	0.01	0.0003
Functional status	-0.006	0.67	0.02	0.32	-0.002	0.90	-0.00006	0.99	0.004	0.67
Working for income	0.2	0.0001	0.1	< 0.0001	0.2	< 0.0001	0.2	< 0.0001	0.2	< 0.0001
Time-varying covariates										
Dialysis (Yes)	-0.3	0.08	-0.6	0.16	-0.2	0.42	-0.09	0.46	-0.09	0.51
Ascites	0.03	0.49	0.01	0.66	0.03	0.29	0.02	0.26	0.02	0.37
Encephalopathy	-0.05	0.28	-0.02	0.51	-0.03	0.27	-0.01	0064	-0.01	0.63
Albumin	0.3	< 0.0001	0.2	0.0006	0.2	< 0.0001	0.2	< 0.0001	0.2	< 0.0001
Bilirubin	-0.05	0.09	-0.07	0.02	-0.08	< 0.0001	-0.09	< 0.0001	-0.1	< 0.0001
Creatinine	-0.06	0.62	-0.1	0.45	-0.4	0.003	-0.4	< 0.0001	-0.4	< 0.0001
INR	-0.2	0.15	-0.4	0.07	-0.4	0.003	-0.4	< 0.0001	-0.4	< 0.0001
Serum sodium	0.02	0.006	0.02	0.0008	0.02	< 0.0001	0.02	< 0.0001	0.02	< 0.0001
Follow-up time	-0.0001	< 0.0001	-0.00008	< 0.0001	-0.00008	< 0.0001	-0.00008	< 0.0001	-0.00009	< 0.0001
